# Health and service utilization among a sample of gender-diverse youth of color: the TRUTH study

**DOI:** 10.1186/s12889-022-14585-9

**Published:** 2022-12-10

**Authors:** Joshua A. Rusow, Marco A. Hidalgo, Sam Calvetti, Meg Quint, Su Wu, Bethany C. Bray, Michele D. Kipke

**Affiliations:** 1 The Brown School, Washington University in St. Louis, St. Louis, MO USA; 2 Gender Health Program, UCLA Health, Los Angeles, CA USA; 3 Department of Medicine, David Geffen School of Medicine, University of California Los Angeles, Los Angeles, CA USA; 4 Children’s Hospital Los Angeles, Los Angeles, CA USA; 5 The Fenway Institute, Fenway Health, Boston, MA USA; 6 Division of Endocrinology, Diabetes and Hypertension, Transgender Health Research, Brigham and Women’s Hospital, Boston, MA USA; 7 Institute for Health Research and Policy, University of Illinois Chicago, Chicago, IL USA; 8 Department of Pediatrics, Keck School of Medicine, University of Southern California, Los Angeles, CA USA

**Keywords:** Mental health, Nonbinary gender identity, Sexually transmitted infections, Substance use, Transgender, Youth, Gender diverse

## Abstract

**Background:**

While there is growing research considering the experiences of transgender youth whose identities align with the gender binary, especially among young trans women, there are significantly fewer studies that accurately capture data about nonbinary youth, and even fewer studies capturing the experiences of transgender and gender diverse (TGD) youth of color. The purpose of this research was to assess the prevalence of sexual health behaviors, mental health challenges, substance use, and healthcare utilization among Black/African American, Latinx, Asian/Pacific Islander, indigenous and multi-racial/ethnic TGD youth, who have been largely underrepresented in research.

**Methods:**

A total of 108 TGD youth ages 16–24 were recruited into the Trans Youth of Color Study (TRUTH). Each participant completed a 90-min survey administered by a research assistant with more sensitive information collected using ACASI. In addition to a completing a survey administered by research staff, participants also participated in specimen collection, which included urine sampling to assess recent substance use without a prescription, self-collected rectal/frontal and throat swabs to test for gonorrhea and chlamydia, and a blood draw to test for recent use of drugs, gonorrhea and chlamydia, and syphilis. The sample was recruited at public venues, community outreach and referral, through social media outreach, and via participant referral. Cross-sectional analyses were from a single study visit.

**Results:**

Compared to rates among their cisgender peers, participants reported experiencing adverse social and structural determinants of health—e.g. food insecurity (61%), housing instability (30%), and limited access to healthcare (26% had no place to go for healthcare)—and elevated rates of illicit drug use (19–85%), mental health problems (e.g. 60% self-reported depression), and involvement in sexual risk-related behaviors (e.g. among those reporting penetrative sex 57–67% reported sex without a condom).

**Conclusions:**

This study adds descriptions of both mental and sexual health outcomes of a non-clinical sample of TGD youth to the literature, particularly among young transgender men and gender nonbinary youth, who have frequently been excluded from previous studies of sexual health. The findings document experiences and behaviors among TGD youth that contribute to mental and sexual health concerns, including rates of substance use, and healthcare utilization.

**Supplementary Information:**

The online version contains supplementary material available at 10.1186/s12889-022-14585-9.

## Background

Recent estimates suggest over 300,000 individuals ages 13–17 and over 1.6 million individuals 13 years or older in the United States (U.S.) are transgender or gender diverse (TGD) [1]. Among U.S. adolescents ages 13–17 years old, approximately 1.3, 1.4, 1.0, 1.8, 1.8, and 1.5 percent of white, Black, Asian, American Indian and Alaska Native, Latinx, and other races respectively are TGD, indicating the importance of capturing racially and ethnically diverse experiences in TGD health research [1]. TGD people are those whose gender identity and/or expression does not align with their sex assigned at birth [2]. While most TGD people—not necessarily including TGD intersex people—have similar experiences in that their gender identity does not align with their sex assigned at birth, the “trans” community is not a monolith as some individuals interact with and others completely reject the gender binary. Whereas binary-aligned TGD individuals may possess a gender identity that aligns largely within cultural norms associated with a man-woman binary (e.g., using labels such as man, woman, trans woman, trans man, and often pronouns such as “he/him”, “she/her”), nonbinary TGD individuals may fall outside a man-woman or a masculine-feminine binary (e.g., using labels such as nonbinary, agender, and gender-neutral pronouns such as “they/them”, “ze”, “zir” or one’s name in place of a pronoun) [3]. Among binary-aligned transgender people, trans man usually refers to those who were assigned female sex at birth but identify and/or present as a man, while trans women are those who were assigned male sex at birth but identify and/or present as a woman. Nonbinary, gender non-conforming, genderqueer, agender, and other gender expansive identities (referred to as nonbinary for the purposes of this paper) represent those who either exist outside of or completely reject the gender binary [4, 5]. Nonbinary individuals may or may not categorize themselves as part of the larger transgender community and may experience unique stressors due to existing in a system that upholds a gender binary [6, 7]. Due to a lack of historical representation of nonbinary gender designations within research studies, much of the existing literature around transgender individuals categorizes them into binary-aligned groups.

TGD youth (inclusive of adolescents and emerging adults) are at disproportionally high risk for acquiring sexually transmitted infections (STIs) [8–11] and developing mental health conditions compared with rates in heterosexual and presumably cisgender samples where gender history was not collected [12–14]. Without proper measures in probability samples, it is hard to estimate the number of nonbinary youth, however in a community sample of TGD adults measured in 2015, over one-third of respondents primarily identified as nonbinary [15], and in a clinical sample of TGD adolescents, 9% identified as nonbinary [16]. Among lesbian, gay, bisexual, transgender, queer, questioning, plus (LGBTQ +) adults, nonbinary people are more likely to identify as cisgender (58%) than transgender (42%), however, nonbinary adults make up more of the transgender LGBTQ + adult population (32%) than the cisgender LGBQ adult population (8%) [17]. The vast majority of research on TGD individuals derives from samples of binary-aligned individuals in the context of human immunodeficiency virus (HIV)/STI prevention (e.g., trans women, trans men). This extant literature has underscored the high and disproportionate concentration of HIV/STI prevalence among adult and adolescent transgender populations, namely among those most researched; Black/African American and Latinx trans women and trans men who have sex with cisgender men [18–23]. From these studies have emerged HIV prevalence estimates among trans women as high as 44% and 26% in African American/Black and Latinx adults, respectively [24].

In the limited studies that capture the expansive experience and identities of TGD youth, we find that nearly one-third of TGD youth are at a high risk for acquiring STIs [11]. One study of 145 sexually active transgender adolescents in urban New England found that nearly half the sample had engaged in either or both condomless insertive vaginal (frontal) and insertive/receptive anal intercourse, although context of the sexual encounters including relationship status and pregnancy intentions were not included [25]. Another study of young transgender women found that 49% had engaged in recent condomless receptive anal intercourse, and 53% had sex under the influence of drugs or alcohol [19]. Other researchers have also reported similar incidence of STIs for binary-aligned TGD youth (both transfeminine and transmasculine), however differences were observed by gender across the specific STIs assessed, highlighting the need for a detailed accounting of the experiences of nonbinary TGD youth [25].

Compared to their cisgender counterparts, TGD youth are at a disproportionately high risk for negative mental health outcomes and illicit drug use [26–28]. TGD adolescents are three to six times more likely to attempt suicide than their cisgender peers [29]. Another study found that more than 50% of the transmasculine youth, 40% of nonbinary youth, and 30% of transfeminine youth reported a suicide attempt over a 36-month period [30]. College-age nonbinary individuals reported higher levels anxiety, depression, psychological distress, and eating concerns than did binary-aligned TGD and cisgender individuals [31]. Among transgender adults in the United States surveyed online in 2015, 40% reported having attempted suicide in their lifetime [15]. Many studies, particularly through 2015, focus on transgender men and women and fail to access the experiences of nonbinary youth [32]. Most of these studies either inaccurately classify nonbinary individuals as transmasculine or transfeminine due to data collection methods or fail to include nonbinary individuals entirely. Little is known about the drug use behaviors of TGD youth; however, a recent study found that among transfeminine youth, drug use was associated with post-traumatic stress disorder, gender-related discrimination, and psychological distress [33]. Relative to cisgender adolescents, TGD adolescents in California were 2.5 to 4 times more likely to report substance use and began using substances at an earlier age [26]. One study found that rates of mental health problems, self-harm, and suicidality, and alcohol use were elevated in both binary and nonbinary individuals, but indicated important differences by both sex assigned at birth and binary/nonbinary status, including assigned female at birth participants reporting a higher rate of mental health conditions than assigned male at birth [34].

Due to historic stigmatization, pathologizing, and discrimination by mental and physical health professionals, TGD youth may be less likely to seek out services meant to address substance use, psychological distress, or sexual health and wellness. Many TGD youth do not share their gender or sexual identity with their providers for fear of discrimination or being outed, which makes them less likely to receive adequate sexual health information or care [35]. Among adults, transgender individuals have lower rates of healthcare utilization and high rates of avoidance due to anticipated discrimination, though this has yet to be adequately evaluated in younger populations who might also need to consider the complexities of privacy and being on a parent’s insurance plan [36–39]. A recent study found that nonbinary patients experience higher rates of disrespect from providers and indicated an association between depression or suicidal thoughts and having the burden of educating providers [40]. Among a sample of TGD patients receiving care in Austria, 60.5% of TGD participants reported not being taken seriously in healthcare settings and nonbinary patients had worse patient-physician relationships than trans male patients [41]. Specifically, systemic bias within the healthcare system is well documented in transgender populations, leading to disparities in access, mistreatment in healthcare settings, outright refusal of care by health providers, and postponement of care [15, 42]. The limited research on healthcare access for nonbinary adults suggests that this group encounters barriers to care and health disparities at even higher rates than binary transgender adult populations [43]. Nonbinary youth may, therefore, benefit from targeted interventions that account for their higher risk for a range of poor health outcomes, and because of barriers they face in accessing necessary HIV prevention, testing, treatment, and other healthcare services.

The TGD community is a diverse community of people who often hold multiple marginalized and intersecting identities. Previous research has indicated exceptionally high rates of HIV in TGD women of color [21, 44] and higher rates of substance use and suicide attempts than white TGD people [15]. TGD people of color describe how they must consider both race and TGD identities when seeking care as TGD competent providers still may hold racist views and vice versa [45]. There continues to be a great need and opportunity for research that explores the intersection of race/ethnicity and gender identity.

To expand the literature exploring the unique experiences and differential health outcomes between trans and nonbinary identified youth, this study evaluates the various physical, emotional, and behavioral outcomes between binary-aligned TGD youth and nonbinary TGD youth in a racially and ethnically diverse, community-based sample from Los Angeles County. With the aim of underscoring the unique healthcare needs of the diverse group that is the TGD community, key outcomes include rates of STIs, mental health outcomes, chronic health conditions, substance use, and healthcare and service utilization.

## Methods

### Study design

The purpose of this research was to assess the sexual health behaviors, mental health challenges, substance use, and healthcare utilization experiences of Black/African American, Latinx, Asian/Pacific Islander, indigenous and multi-racial/ethnic TGD youth. A total of 108 TGD youth were recruited into the Trans Youth of Color Study (TRUTH). Each participant completed a 90-min survey administered by a research assistant with more sensitive information collected using ACASI. In addition to completing a survey administered by research staff, participants also participated in specimen collection, which included urine sampling to assess recent substance use without a prescription, self-collected rectal/frontal and throat swabs to test for gonorrhea and chlamydia, and a blood draw to test for recent use of drugs, gonorrhea and chlamydia, and syphilis [46]. Cross-sectional data in this study were collected from participants during a single study visit. A Youth Community Advisory Board (YAB) and Community Advisory Board (CAB) were initially established to provide guidance regarding the methods used to conduct the research, including refinement of the study questions, survey instrument, recruitment design, and analysis, interpretation, and dissemination of the study findings. The YAB consisted of TGD participants from a similar study [46], as well as other TGD youth that they personally recommended. The CAB was formed by inviting members of the CAB from one of our previous studies [46] and community stakeholders from longstanding Los Angeles County transgender health organizations and providers to an open house to learn about the project and to solicit suggestions about study procedures. This study received Institutional Review Board approval, and a Certificate of Confidentiality was also obtained from the National Institute on Drug Abuse.

### Participants

Youth were eligible to participate in the study if they met the following criteria: (1) self-identify as Black/African American, Latinx, and another or multi-racial, including Asian/Pacific Islander and indigenous; (2) self-identified as TGD (e.g., transgender, nonbinary, identified differently than their sex designated at birth); (3) were 16- to 24-years of age; (4) were English- or Spanish-speaking; and (5) resided in Los Angeles County. A waiver of parental consent was obtained for participants 16–17 years old. A brief screening interview was used to determine eligibility. Those who met study eligibility criteria and volunteered to participate were provided a brief study description and asked to provide contact information. An in-person follow-up consent appointment was then scheduled within one week of recruitment, where research staff provided detailed information about the study—through a set of infographics and a printed information sheet—and obtained informed consent/assent. Participants could meet with research staff at the study research offices, or a location more convenient to the participant if the alternate location was both safe and private. All participants provided written informed consent during the face-to-face visit consenting visit. Participants received a $65 incentive as compensation for their time and effort. All consent procedures, incentive amounts, and study procedures were approved by the Children’s Hospital Los Angeles Institutional Review Board (IRB# 14–00279).

### Recruitment

Participants were recruited using a range of strategies adapted from another study and informed by our YAB and CAB [46], including (1) direct outreach at and referrals from community-based organizations and at public spaces frequented by our targeted population, such as Pride festival, parks and street corners (used to recruit 20% of the sample); (2) through social media (used to recruit 56% of the sample), and (3) participant referral (used to recruit 24% of the sample). The following provides a description of our recruitment strategies.

#### Direct outreach through LGBTQ + youth-serving agencies, locations and events

Youth were recruited from public venues, including LGBTQ + youth- and TGD youth-serving community-based organizations and social service agencies and materials distributed to student groups at high schools and universities. Participants were also recruited using at street locations where TGD youth congregated and LGBTQ + special events such as LGBTQ + Pride festivals. Of the 79 people who preliminarily screened eligible through direct outreach, 21 (27%) enrolled into the study. Twenty percent of the sample was recruited through these direct methods.

#### Social media and online recruitment

TGD youth were also recruited using social media advertising on Instagram and Facebook, with advertisements also being posted on Craigslist and disseminated by local LGBTQ + college and high school listservs. A total of 156 individuals responded to advertisements, of which 84 (54%) met the study eligibility criteria and 61 (40%) agreed to participate in the study. Fifty-six percent of the sample was recruited through social media and online outreach.

#### Referral recruitment

Study participants could refer up to two friends to participate in the study; participants were paid $10 for each eligible friend who consented or assented (for participants younger than 18 years old) to participate in the study. Of the 14 participants that referred others to the study, the average number of referrals who enrolled in the study was 1.71. Twenty-four percent of the sample was recruited through participant referrals.

### Measures

Study participants completed a 90-min survey administered by research staff with measures about sexual activity, substance use, suicidality, and gender-based discrimination contained within self-administered sections to provide additional privacy.

#### Demographic characteristics

Data collected included gender identity, race and ethnicity, age, sex assigned at birth, residential status, education/employment, food security/hunger, having money to cover basic needs, sexual attraction, history of sex exchange, experiences in foster care and incarceration.

#### Alcohol, tobacco, marijuana, and illicit drug use

Scales from the Monitoring the Future and 2014 National Survey on Drug Use and Health studies were used to assess lifetime, past 6-month and past 30-day substance and alcohol use [47, 48]. Substances included alcohol, tobacco, marijuana, cocaine, crack, heroin, ecstasy, methamphetamines, GHB, ketamine, poppers, inhalants, hallucinogens, and prescription drugs used without a physician’s order. Participants were asked if they were actively being prescribed any substances categorized within this list. Urine samples tested for metabolites of amphetamines, methamphetamines, benzodiazepines, cocaine, ecstasy, phencyclidine, methadone, fentanyl, marijuana, and opiates using the Integrated E-Z Split Key Cup II- 10 Panel (Innovacon Laboratories), which can detect drugs one to four days after use, except for chronic marijuana use, which can be detected for up to 30 days [49]. Screening for fentanyl was also performed.

#### Problem alcohol and marijuana use

The Alcohol Use Disorders Identification Test (AUDIT) was used to assess the severity/frequency of participants’ alcohol use [50, 51]. Problems resulting from marijuana use were measured by 13 items based on the Diagnostic and Statistical Manual of Mental Disorders (DSM)-IV [52] and DSM-5 [53] criteria for abuse, dependence, or disorder.

#### STI/HIV history, test results, and prevention behaviors

Participants self-reported any STI testing history and test-positivity count [46]. Specimen self-collection assessed for Neisseria gonorrhea (urine, rectal/vaginal [frontal], and pharyngeal specimens) and Chlamydia trachomatis (urine and rectal/vaginal [frontal] specimens) using a nucleic acid amplification test. Syphilis testing was performed using whole blood collected via venipuncture using a rapid plasma reagin and treponemal antibody test. HIV testing was performed using a 4^th^ generation point-of-care rapid whole blood finger-stick HIV test (Alere, Inc., Waltham, MA), a Food and Drug Administration (FDA)-approved diagnostic measure of HIV-1 p24 antigen and HIV-1/2 antibodies. Those with positive results met with the on-site STI/HIV test counselor, then were referred to treatment at one of our partner clinical sites.

#### Sexual risk behaviors, partners and HIV risk, and protective behaviors

Sexual risk behaviors, partners and HIV risk, and protective behaviors were assessed using scales adapted from a previous study conducted with young men who have sex with men to be more inclusive of TGD experiences [54, 55]. Input from our YAB members and CAB partnerships allowed us to adapt this measure to include sexual activity categorized by specific body part interactions (i.e. Your Penis in Their Vagina). Participants were asked about their *lifetime and recent sexual experiences* (past 30 days and 6 months), including insertive/receptive oral, frontal, and anal sex. Participants were also asked the frequency they engaged in each type of sexual activity and the gender of their partners, each type of sexual activity for *different partner types* (primary, consistent casual, casual) in the past 6 months, and *frequency of condom* use by gender of partner and by sexual activity type. Participants were also asked if they had ever/recently (past 6 months) exchanged sex for money, drugs, or other needs.

#### Measures of overall health, mental health, and healthcare utilization

Using modified questions from the Youth Risk Behavior Survey [56], participants were asked about their overall health status, anxiety about their health, and whether they had a chronic health condition. Participants’ access to and use of the healthcare system, including insurance, was measured using survey questions from the National Longitudinal Study of Adolescent to Adult Health Study and the National Survey of Children’s Health [57, 58]. The 18-item Brief Symptom Inventory (BSI) was used to assess depression, anxiety, and somatic complaints, as well as lifetime and current suicidality and self-injurious behavior [59].

### Data analysis

Descriptive statistics and corresponding statistical tests for group comparisons were calculated for demographic characteristics, substance use, health, mental health and healthcare experiences. Most variables were categorical and frequencies of responses for the overall sample and by gender identity are presented in Tables 1, 2, 3, 4, 5. Given limited research on nonbinary youth, group comparisons for binary-aligned (i.e. trans women and trans men) vs. nonbinary (including nonbinary, gender non-conforming, genderqueer, or another non-cisgender identity) participants were explored using Pearson’s chi-square tests. Gender identity was collapsed into binary-aligned transgender participants and nonbinary participants, both as a means to detect differences between these distinct groups and pragmatically to ensure there were enough participants in each group to detect differences. Means, standard deviations, and overall *F*-tests from one-way analyses of variance tests are presented for age, the only continuous variable, of which the sample skewed older.

## Results

### Demographic characteristics

As presented in Table 1, 79% of the sample was designated female at birth, 65% reported a nonbinary gender identity (examples are: gender nonbinary, agender, genderqueer, gender fluid, gender non-conforming, or other nonbinary identities) and 35% reported binary-aligned gender identities. The mean age was 21.4 years and 65% were 21 to 25 years old. Thirty-nine percent identified as Latinx, 18% as Black/African American, and 43% multi-racial/ethnic or another racial/ethnic identity including Asian/Pacific Islander or indigenous. Although 45% reported living with their family, binary-aligned TGD youth were significantly more likely than nonbinary TGD youth to have no regular place to live and less likely to live in their own place (*p* < 0.02). Participants experienced considerable challenges making ends meet, with 61% reporting food insecurity or hunger in the previous 12 months and 69% having run out of money and unable to meet their basic needs at least once in the previous 3 months. While 54% of participants reported full-time or part-time work, 33% reported that they were not working but looking for employment. In the past 6 months, overall, 57% had been in a primary partner relationship and 29% had engaged in sex work.

**Table 1:** TRUTH Cohort – Demographic characteristics by gender identity (*N* = 108)

Variable	Categories	TGD^a^ Youth Total (*N* = 108)	Binary TGD Youth (*n* = 38)	Nonbinary TGD Youth (*n* = 70)	*P*-value
Race n (%)	Black only Latinx only Multi-Racial/Ethnic and Other identities^b^	19 (18) 42 (39) 47 (43)	8 (21) 18 (47) 12 (32)	11 (16) 24 (34) 35 (50)	0.18
Age	Mean (SD^c^)	21.39 (2.09)	21.03 (2.31)	21.58 (1.95)	0.19
Age Category n (%)	< 18 years 18 – 20 years 21 – 25 years	8 (7) 30 (27) 70 (65)	3 (8) 15 (40) 20 (53)	5 (7) 15 (21) 50 (71)	0.12
Sex Designated at Birth n (%)	Male Female	23 (21) 85 (79)	8 (21) 30 (79)	15 (21) 55 (79)	0.96
Residential Status n (%)	Family Own place/apartment With friends/partner No regular place/other	49 (45) 27 (25) 14 (13) 18 (17)	14 (37) 7 (18) 5 (13) 12 (32)	35 (50) 20 (29) 9 (13) 6 (9)	**0.02**
Education n (%)	Less than high school^d^ High school/GED Vocational school & some college AA to Bachelor & beyond	16 (15) 26 (24) 51 (48) 14 (13)	9 (24) 13 (34) 14 (37) 2 (5)	7 (10) 13 (19) 37 (54) 12 (17)	**0.02**
Employment Status n (%)	Yes, Part-time Yes, Full-time Not working & not looking Not working & looking	41 (38) 17 (16) 14 (13) 35 (33)	14 (38) 5 (13) 7 (19) 11 (30)	27 (39) 12 (17) 7 (10) 24 (34)	0.61
Food Security n (%)	Food security Food insecurity Hunger	42 (39) 25 (23) 41 (38)	17 (45) 11 (29) 10 (26)	25 (36) 14 (20) 31 (44)	0.18
Basic Needs n (%)	Did not run out money 1–3 times a month 1 + a week	33 (31) 57 (53) 16 (15)	13 (35) 18 (49) 6 (16)	20 (29) 39 (57) 10 (15)	0.73
Primary partner (last 6 months) n (%)	Yes	61 (57)	18 (72)	43 (71)	0.89
Sex Exchange (last 6 months) n (%)	Yes	31 (29)	13 (34)	18 (26)	0.38
History of foster care n (%)	Yes	8 (7)	5 (13)	3 (4)	0.13
History of incarceration n (%)^h^	Yes	5 (5)	2 (5)	3 (4)	0.58

^a^*TGD* transgender and gender diverse

^b^Other identities include Asian/Pacific Islander and Indigenous

^c^*SD* standard deviation

^d^6 participants who reported “Less than high school” were ages 19 or older (4 binary TGD; 2 nonbinary)

^e^Participants were not asked to differentiate between cisgender men and trans men

^f^Participants were not asked to differentiate between cisgender women and trans women

^g^Participants were not asked to differentiate between trans women, trans men, or nonbinary partners

^h^Ever been sent to jail or prison for any reason

### Sexual behaviors that can lead to STI exposure

Reports of sexual positioning and condom use are presented in Table 2. During the past 6 months, 60% of nonbinary participants designated *male* at birth reported having engaged in both insertive and receptive anal intercourse whereas 27% and 33% of nonbinary participants designated *female* at birth reported having engaged in receptive anal intercourse and receptive frontal intercourse, respectively. Among binary-aligned trans men, 21% reported receptive anal intercourse and 17% reported receptive frontal intercourse during the past 6 months. Among binary-aligned trans women participants, 56% reported receptive anal intercourse during the past 6 months. Of those who reported sexual intercourse, 71% of binary-aligned trans men, 100% of binary-aligned trans women, and 65% of nonbinary participants reported not using a condom or not being sure if a condom was used during their last anal or frontal sexual encounter.

**Table 2:** Sexual activity and condom use by designated sex and gender identity in last 6 months

	**Designated Male at Birth (*n* = 24)**		**Designated Female at Birth (*n* = 84)**	
	**Binary TGD Youth (*n* = 9)**	**Nonbinary TGD Youth (*n* = 15)**	**Binary TGD Youth (*n* = 29)**	**Nonbinary TGD Youth (*n* = 55)**
Anal Insertive Sex n(%)				
Yes	5 (56)	9 (60)	3 (10)	10 (18)
Anal Receptive Sex n(%)				
Yes	5 (56)	9 (60)	6 (21)	15 (27)
Frontal Insertive Sex n(%)				
Yes	3 (33)	7 (47)	14 (48)	32 (58)
Frontal Receptive Sex n(%)				
Yes	1 (11)	1 (7)	5 (17)	18 (33)
Condomless Sex during most recent sexual encounter n(%)				
Yes	4 (67)	8 (57)	11 (65)	32 (67)
Unsure	2 (33)	0 (0)	1 (6)	0 (0)

Note: Sexual behaviors reported here involve a flesh penis. Condomless sex was assessed among those reporting any insertive or receptive sex during the past 6 months. *TGD* transgender and gender diverse

## Mental health

Although 85% of participants reported having wanted or needed mental health counseling during the previous 12 months, only 30% were able to obtain these services (Table 3). More nonbinary than binary-aligned participants reported needing or wanting services during the past 12 months (*p* = 0.03). In addition, 13% of TGD youth scored in the clinically-significant range for depression, 14% for anxiety, and 10% for somatization on the BSI. Self-injury was reported by 76% of participants—29% having done so in the past 3 months. Suicidal ideation was reported by 54% of participants and 15% had attempted suicide during the previous 12 months.

**Table 3:** Mental health by gender identity

**Variable**	Categories	Total (*N* = 108) n (%)	Binary TGD Youth (*n* = 38) n (%)	Nonbinary TGD Youth (*n* = 70) n (%)	*P*-value
BSI^a^ – Scored in clinically significant range (last 7 days) n(%)	Depression Anxiety Somatization	14 (13) 15 (14) 11 (10)	3 (8) 2 (5) 3 (8)	11 (16) 13 (19) 8 (11)	0.37 0.08 0.74
Needed/wanted mental health services (past 12 months) n(%)	Yes	92 (85)	29 (76)	63 (91)	**0.03**
Received mental health services (past 12 months) n(%)	Yes	28 (30)	11 (38)	17 (27)	0.29
Suicide (past 12 months) n(%)	None Ideation Made a plan Attempted	43 (40) 51 (54) 36 (37) 16 (15)	20 (57) 15 (43) 10 (29) 5 (13)	23 (39) 36 (61) 26 (41) 11 (16)	0.09 0.09 0.21 1.00
Self-injurious behavior n(%)	Ever Last 3 months	73 (76) 28 (29)	24 (75) 7 (22)	49 (77) 21 (33)	0.87 0.27

^a^*BSI* Brief Symptom Inventory

### Tobacco, alcohol, marijuana, and illicit drug use

As presented in Table 4, the majority of participants reported both lifetime and past 6 month use of alcohol (90% and 81%, respectively), tobacco (67% and 46%, respectively), and marijuana (85% and 76%, respectively). Thirty-seven percent had used hallucinogens (10% in the past 6 months), 26% reported cocaine use (15% in the past 6 months), 21% reported ecstasy use (7% in the past 6 months), 19% reported poppers use (12% in the past 6 months), and 23% some other illicit drug (e.g., methamphetamine, GHB; 7% in the past 6 months). Nearly half of the sample (48%) tested positive for using one or more illicit drugs. No differences in substance use were observed by gender identity.

**Table 4:** Substance use by gender identity

Variables	Categories	Total (*N* = 108) *n* (%)	Binary TGD Youth (*n* = 38) *n* (%)	Nonbinary TGD Youth (*n* = 70) *n* (%)	*P*-value
Alcohol	Lifetime 6 months	97 (90) 87 (81)	33 (87) 27 (71)	64 (91) 60 (86)	0.51 0.07
Tobacco	Lifetime 6 months	72 (67) 50 (46)	23 (61) 14 (37)	49 (70) 36 (51)	0.32 0.15
Marijuana (Non-Rx)	Lifetime 6 months	92 (85) 82 (76)	30 (79) 26 (68)	62 (89) 56 (80)	0.18 0.18
Poppers	Lifetime 6 months	20 (19) 13 (12)	4 (11) 4 (11)	16 (23) 9 (13)	0.13 1.00
Cocaine	Lifetime 6 months	28 (26) 16 (15)	7 (18) 3 (8)	21 (30) 13 (19)	0.19 0.14
Ecstasy	Lifetime 6 months	23 (21) 8 (7)	6 (16) 5 (13)	17 (24) 3 (4)	0.34 0.13
Hallucinogen	Lifetime 6 months	40 (37) 11 (10)	11 (29) 2 (5)	29 (41) 9 (13)	0.20 0.32
Other drugs used (Non-Rx)^a^	Lifetime 6 months	25 (23) 8 (7)	11 (29) 2 (5)	14 (20) 6 (9)	0.29 0.71
Positive drug test ^b^	0 Drugs 1 + Drug	56 (52) 42 (48)	21 (55) 12 (32)	35 (50) 30 (43)	0.39
Alcohol use perceived a problem (last 6 months) ^c^	No Needed advice Needed counseling Needed evaluation for dependence	73 (68) 28 (26) 5 (5) 2 (2)	31 (82) 5 (13) 1 (3) 1 (3)	42 (60) 23 (33) 4 (6) 1 (1)	0.06
Marijuana use perceived a problem (last 6 months) ^d^	No problems Mild problems Moderate problems Severe problems	31 (38) 23 (28) 21 (26) 7 (9)	10 (39) 8 (31) 6 (23) 2 (8)	21 (38) 15 (27) 15 (27) 5 (9)	0.98

^a^ Other Non-Rx drugs include fake marijuana, heroin, fentanyl, meth, GHB, ketamine, inhalants, and others

^b^ Missing data were excluded from calculations. There was a total of 10 participant whose drug test results were missing

^c^ The “No” category includes 21 participants who said they hadn’t drunk alcohol in the last 6 months

^d^ 26 participants who said they hadn’t used marijuana in the last 6 months were excluded from calculations

Regarding alcohol problems indicated by the AUDIT, 26% needed simple advice and 7% needed brief counseling or evaluation for alcohol dependence. Similarly, 35% scored in the moderate to severe problem range for marijuana use. There were no significant differences by gender identity or race/ethnicity for alcohol or marijuana use.

### Healthcare utilization and diagnoses

While most participants reported having insurance (91%) and a place to go for healthcare (74%), 63% reported being worried about their health. Nonbinary participants were significantly more likely to report being worried about their health (*p* < 0.01). Participants self-reported their diagnoses of physical or mental illnesses. Seventy-nine percent of participants self-reported at least one chronic health condition, most commonly depression (60%), anxiety (56%), asthma (21%), or post-traumatic stress disorder (PTSD; 20%).

Over one-third of the sample (39%) reported having previously been diagnosed with an STI; 6% tested positive for one or more STIs at time of their assessment (Table 5). No HIV-negative participants tested positive for HIV during the visit. Three (3%) TGD youth were currently taking pre-exposure prophylaxis (PrEP) for HIV prevention and eight (8%) had ever taken PrEP. Additionally, two HIV-positive participants were enrolled in the TRUTH cohort.

**Table 5:** Health, healthcare, and STIs by gender identity

Variable	Categories	Total (*N* = 108) n (%)	Binary TGD Youth (*n* = 38) n (%)	Nonbinary TGD Youth (*n* = 70) n (%)	*P*-value
Insured n(%)	Yes	97 (91)	36 (95)	61 (88)	0.28
Source of insurance^**a**^ n(%)	No Health Insurance Public Health Insurance Private Health Insurance	9 (8) 46 (43) 52 (49)	2 (5) 21 (55) 15 (40)	7 (10) 25 (36) 37 (54)	0.15
Have a place to go for healthcare n(%)	Yes	80 (74)	30 (79)	50 (71)	0.39
Worried about health n(%)	Yes	65 (63)	17 (46)	48 (73)	**0.01**
Perception of overall health^**a**^ n(%)	Poor Fair Good Very Good Excellent	11 (11) 37 (36) 35 (34) 15 (14) 6 (6)	2 (6) 13 (37) 11 (31) 5 (14) 4 (11)	9 (13) 24 (35) 24 (35) 10 (15) 2 (3)	0.42
Chronic health condition n(%)	Yes	85 (79)	32 (84)	53 (76)	0.34
Most common chronic health conditions n(%)	Depression Anxiety Asthma PTSD	65 (60) 60 (56) 23 (21) 22 (20)	22 (58) 20 (53) 13 (34) 7 (18)	43 (61) 40 (57) 10 (14) 15 (21)	0.72 0.65 **0.02** 0.71
Self-Report history of STIs^**b**^^**,c**^ n(%)	1 + STIs	29 (39)	11 (41)	18 (38)	0.78
Test positive for STIs^**b**^^**,d**^ n(%)	1 + STIs	6 (6)	4 (11)	2 (3)	0.18

^a^Those who answered “don’t know” or refused to answer were excluded from analyses

^b^STIs = sexually transmitted infections

^c^33 (31%) individuals did not test, or were not sure they had tested for STIs

^d^8 (7%) individuals did not have any STI lab test results

## Discussion

These findings expand upon the limited research among TGD youth of color, particularly nonbinary and transmasculine youth. In this paper, we describe an ethnically and racially diverse sample of TGD youth, an important population that has been largely neglected within the research literature. Our findings indicate high rates of suicidality, depression, and other mental health conditions among the TGD youth in this study. This aligns with prior studies among TGD youth have found that, compared to cisgender youth, TGD youth experience two to three times the rates of depression [28, 60], suicidal ideation [28, 29, 60], self-harm [28, 60], and anxiety [28]. Reporting on and encouraging expansive reporting of gender within research studies focused on transgender youth will allow an increasing knowledgebase of specific difficulties nonbinary youth face. Other projects allowing for this distinction among genders plus our findings expand on the challenges faced by this group of young people in behavioral health concerns and accessing care [34, 40, 41, 61].

The complex relationships between social and structural barriers, experiences of racism, transphobia, and other stressful life events and their intersectional impact on TGD youth health have begun to be explored in the literature [35–38]. As noted in previous research, the participants in this sample reported difficulties accessing health and mental health care that they felt they needed [61]. We observed high rates of hunger and financial insecurity in this sample, as well as reports of sex exchange. Although we could not look at associations between these experiences and health outcomes, we note the rates of these experiences at young ages and the implications that holding multiple marginalized identities might bear on these experiences. Future research should further explore the intersecting gender and sexual identity spectra alongside the racial and ethnic identity of this population through qualitative or mixed-methods approaches, as well as gender fluidity and identity transition through longitudinal research.

Although none of our participants tested newly positive for HIV, a high percentage reported involvement in behaviors associated with HIV transmission, including condomless sex, exchange sex and substance use. Moreover, despite high rates of eligibility for PrEP, very few reported prior or current use of PrEP. Historically, sexual health education in United States school system vary greatly between state [62] with many states even requiring teachers portray LGBTQ + individuals negatively or preventing discussion of LGBTQ + identities. The inapplicability of school sexual health education leaves youth to seek out education from others like peers and online sources, which still might not be helpful or accurate [63]. Some samples of TGD people, particularly TGD women, have low HIV risk perception and knowledge despite high engagement in high HIV risk behavior [64, 65]. Moreover, PrEP messaging is often geared towards cisgender men who have sex with men. Transgender people may have concerns about interactions between PrEP and hormone regimens, and concerns about the conflation of TGD identitiy with HIV risk instead of a behavior based risk assessment [66, 67]. In additon to structural barriers, these factors may contribute to the low uptake in TGD communities as seen in this sample [66, 67]. These findings and the current state of sexual health education underscore a clear indication for HIV prevention efforts targeting a variety of sexual health behaviors, and mechanisms of risk associated with the intersection of gender and sexual anatomy.

Our findings must be considered in terms of the political landscape of the United States, during a time in which many laws targeting TGD youth are being proposed and enacted. In 2021 alone, over 8 states successfully passed and 28 states proposed anti-transgender legislation [68]. Many of these laws directly impact the ability of TGD individuals to access health services and gender affirming care, examples including criminalizing gender affirming care for youth in Arkansas and an order from the governor of Texas classifying gender affirming care as child abuse [69]. Our data were collected in California where there are many TGD friendly policies [70] and we see high health care utilization in this sample compared to studies with populations in other states [71]. This sample does not represent those who live in states with legislation that limits care access or does not have a medical provider culture that values providing competent care to TGD individuals. Future research should directly investigate the role of local, state, and federal policy on the health and healthcare utilization among gender diverse populations.

It is important to note a number of potential study limitations. First, as a small community sample recruited from public venues, social media, and participant referral in one urban area, study findings may not be representative of the larger Black, Latinx, and multi-racial/ethnic TGD youth population. Although we were able to make significant progress by comparing the experiences and health outcomes of binary-aligned vs. nonbinary TGD youth, due to the sample size we were unable to adequately compare the experiences amongst all specific groups (e.g. trans men, trans women, and nonbinary youth). This is of note as trans individuals who transition within the binary have vastly different experiences in regard to obtaining or losing power and privilege within the context of a society that upholds patriarchal values and masculinity, which may impact health outcomes and is a point for future study. Moreover, sample size prevented us from exploring important racial and ethnic differences or the intersection of race/ethnicity and gender identity at depth. Additionally, our survey was available in English and Spanish, but it was administered in English only (no participants opted for the Spanish language version) and therefore may not reflect the experiences of non-English-speaking TGD youth living in Los Angeles County. Finally, although high-risk populations are found to accurately self-report risk-related behaviors [72] and computer-assisted self-interviewing software likely minimized social desirability by recording answers confidentially [73, 74], self-reported data may, nonetheless, under- or over-estimate the true occurrence of reported behaviors. Despite these limintaitons, the findings suggest that both binary-aligned and nonbinary TGD youth experience substantial mental health concerns and are engaging in higher levels of substance use than their cisgender peers [26, 34]. Their reported sexual activity and lack of use of protective measures such as condoms and PrEP suggests that this is an important population for targeting and designing future HIV prevention interventions.

## Conclusion

Consistent with a growing body of research on TGD youth, findings from this study highlight their considerable risk for a range of health problems, including mental and behavioral health conditions [28, 75–78], substance use [33], and healthcare barriers [78–80]. This sample, while living in an area with relatively supportive policies [70], still demonstrates elevated rates of hunger, financial concerns, and exchange sex. Furthermore, despite the high level of health insurance reported in our sample, and the demand for care, most indicated that they were unable to access the care they wanted—particularly among nonbinary youth. Finally, despite most of the sample qualifying for sexual health interventions like PrEP, very few participants were using such strategies as PrEP or condoms for penetrative sex. Additional research is needed to better understand how protective factors, e.g., social support or positive trans identities, can serve as buffers against these stressors and behaviors. Of particular focus should be the role of policies, given they can act as either protective or harmful by enshrining or restricting access to healthcare for TGD youth. These findings could be used to inform the development of interventions to help ensure these young people have equal opportunities to achieve healthy, happy, and productive lives.

## Supplementary Information

**Additional file 1.**

## Data Availability

The datasets used and analyzed during the current study are available from the corresponding author on reasonable request.

## Abbreviations

ACASI: Audio computer-assisted self-interview
AUDIT: Alcohol Use Disorders Identification Test
BSI: Brief Symptom Inventory
CAB: Community Advisory Board
DSM: Diagnostic and Statistical Manual of Mental Disorders
FDA: Food and Drug Administration
GHB: Gamma hydroxybutyrate
HIV: Human immunodeficiency virus
IRB: Institutional Review Board
LGBQ: Lesbian, gay, bisexual, queer, questioning
LGBTQ+: lesbian, gay, bisexual, transgender, queer, questioning, plus
PrEP: Pre-exposure prophylaxis
PTSD: Post-traumatic stress disorder
STIs: Sexually transmitted infections
TRUTH: Trans Youth of Color Study
TGD: Transgender and gender diverse
U.S.: United States
YAB: Youth Community Advisory Board
