# Supplementary material for: Health and service utilization among a sample of gender-diverse youth of color: the TRUTH study
Source: BMC Public Health. 2022 Dec 10;22:2312. doi: 10.1186/s12889-022-14585-9 (PMC9737736; doi:10.1186/s12889-022-14585-9)
Supplement: Supplementary file 1 — Additional file 1. [file 12889_2022_14585_MOESM1_ESM.docx]

Truth Study Baseline Survey

**Section A. Demographics**

***This section is INTERVIEWER-ADMINISTERED***

**DOB**

1. **What is your date of birth?** _______________________
2. **Which of the following terms is the best fit for your gender right now? (Select all that apply).**

Man (cisgender)

Woman (cisgender)

Transgender or trans woman

Transgender or trans man

Two-spirit

Gender non-conforming

Non-binary

Genderqueer

Gender fluid

Other, specify: __________________

I don’t know

1. **What is the zip code of where you currently live or sleep?** _____________________
2. **What is your primary means of transportation?**

Personal automobile or motorcycle

Friend, relative, or neighbor

Public transportation (bus, metro/train)

Bicycle

Walking

Lyft, Uber, other ride share service

Other ________________________

**SKIP Q.A6, IF RESPONSE TO Q.A5 IS NOT “PERSONAL AUTOMOBILE OR MOTORCYCLE”**

1. **What are the main reasons you do not drive a car or motorcycle? (Please check all that apply.)**

Can’t drive due to a medical/physical condition

Can’t afford a car

Can’t afford gas/insurance

Lost driver’s license

Don’t know how to drive a car

No need, everything I need I can access without a car

Other ______________________________

**SKIP Q.A7, IF RESPONSE TO Q.A5 IS “PUBLIC TRANSPORTATION (BUS, METRO/TRAIN)”**

1. **What are the main reasons you do not use public transportation (bus, metro/train)? (Please check all that apply.)**

No service where I am or where I want to go

Poor connections or transfers

I don’t know how to ride the bus

Limited hours of operation

I don’t feel safe on the bus

I can’t afford it

I don’t know about it

I don’t need it

Other ______________________________

1. **In the past 6 months, how often did you run out of money for your transportation needs (gas money, insurance, bus/train fare)?**

Less than once a month

About once a month

1-3 times a month

Once a week

Many times a week

I did not run out of money for my transportation needs

I don’t know

RF

**Education**

1. **What is the highest level in school that you have completed?**

Never went to school

Less than high school

High school graduate / GED

Vocational school

Some college

Completed an AA degree

Completed a bachelor's degree

Some graduate school

Completed graduate school

RF

**School**

1. **Are you currently in school?**

No

Yes, full-time

Yes, part-time

Enrolled in a program but on a temporary leave of absence

RF

**Employment**

1. **Are you currently employed?**

I’m not working at this time and NOT looking for work

I’m not working at this time and looking for work

Yes, part-time

Yes, full-time

RF

**Residential Status**

1. **Where do you sleep most nights of the week? (CARD 1)**

Biological parent’s / legal guardian's home

Other biological / adoptive family member's home

With House Mother / Father / other Housemember

College campus housing

Own home / apartment (with or without roommate)

Friend's home / apartment

Boyfriend / girlfriend / partner's home /apartment

Hotel / motel

Car

Transitional housing (e.g., Covenant House, PATH)

Don't have a home or regular place to stay

Other

DK

RF

**Residential Cross-Streets**

1. **Can you give me the cross streets and/or city of where you sleep most nights of the week?** _____________________
2. **Has there ever been a time when you didn’t have a place to sleep?**

No

Yes

Don’t know

**Now we would like to ask about some of the other places you may have lived during your lifetime.**

| **Have you ever lived or stayed…** |  | **[IF YES] age first time lived there** | **[IF YES] Number of different times lived there:** |
| --- | --- | --- | --- |
| 1. **In a foster home?** | No Yes |  |  |
| 1. **In a group home?** | No Yes |  |  |
| 1. **In juvenile hall or youth camp?** | No Yes |  |  |
| 1. **In a psychiatric hospital?** | No Yes |  |  |
| 1. **In prison or jail?** | No Yes |  |  |

**Ethnicity**

1. **What is your ethnicity? Check all that apply: (CARD 2)**

African

American Indian / Native American

Armenian

Asian / Asian American

Belizean

Black / African American

Caribbean

Central American

Chicano

Chinese (mainland or Taiwan)

Cuban

Dominican

European

Haitian

Hispanic

Japanese

Korean

Latino

Mestizo

Mexican

Middle Eastern/Arab

Pacific Islander

Persian

Pilipino

Puerto Rican

Salvadoran

South American

South Asian

Vietnamese

White / Caucasian

Other: __________________

**Ethnicity (primary)**

1. **Which ethnic group do you identify with most?**

American Indian / Alaska Native

Asian

Black / African American

Native Hawaiian or other Pacific Islander

Hispanic or Latino

White

Mixed

Other: __________________

1. **Where were you born?**

United States

Outside of the United States

I don’t know

Refuse

**Primary language**

1. **In the home you live in now, do you speak:**

Only English

Mostly English

English and another language equally

Mostly another language

Only another language

I don’t know

RF

1. **When you are with your friends, do you speak:**

Only English

Mostly English

English and another language equally

Mostly another language

Only another language

I don’t know

RF

**Financial Hardship**

1. **In the last 3 months, how often did you run out of money for your basic needs (e.g., food, rent, electricity/gas)? (CARD 3)**

I did not run out of money for my basic needs

Less than once a month

About once a month

1-3 times a month

Once a week

Many times a week

I don’t know

RF

**Sexual Identity**

1. **What would you say is your sexual identity or orientation? (CARD 4)**

Gay

Lesbian

Heterosexual (straight)

Bisexual

Queer

Same gender-loving

Downe

Pansexual

Asexual

Demisexual

Unsure / questioning

Other: ___________________

I don’t know

RF

**Attraction to men**

1. **How much are you sexually attracted to men?**

Not at all

Not very strongly

Somewhat strongly

Very strongly

I don’t know

RF

**Attraction to women**

1. **How much are you sexually attracted to women?**

Not at all

Not very strongly

Somewhat strongly

Very strongly

I don’t know

RF

**Attraction to people who are transgender, gender nonconforming, or non-binary**

1. **How much are you sexually attracted to people who are transgender, gender nonconforming, or non-binary?**

Not at all

Not very strongly

Somewhat strongly

Very strongly

I don’t know

RF

**Food security**

These next questions are about the food eaten in your household in the last 12 months and whether you were able to afford the food you need.

1. **“The food that (I/we) bought just didn’t last, and (I/we) didn’t have money to get more.”
    Was that often, sometimes, or never true for (you/your household) in the last 12 months?** Never true

Sometimes true
 Often true
 I don’t know
 Refuse to answer

1. **“(I/we) couldn’t afford to eat balanced meals.” A balanced meal includes fruits, vegetables, whole grains, dairy, and meats/protein. Was that often, sometimes, or never true for (you/your household) in the last 12 months?**
    Never true

Sometimes true
 Often true
 I don’t know
 Refuse to answer

1. **In the last 12 months, did (you/you or other adults in your household) ever eat less or skip meals because there wasn't enough money for food?** No

Yes
 I don’t know

**SKIP Q.A33, IF RESPONSE TO Q.A32 IS “NO” OR “I DON’T KNOW”**

1. **How often did this happen?** Only 1 or 2 months
    Some months but not every month
    Almost every month
    I don’t know
2. **In the last 12 months, did you ever eat less than you felt you should because there wasn't enough money for food?**
    No

Yes
 I don’t know

1. **In the last 12 months, were you every hungry but didn't eat because there wasn't enough money for food?**
    No

Yes
 I don’t know

**Section B. Spiritual Well-Being and Wellness**

***This section is INTERVIEWER-ADMINISTERED***

**Religiosity**

1. **What is your current religion or belief system? Please circle all that apply. (CARD 5)**

Agnostic

Atheist

Baptist

Buddhist

Candomble

Catholic

Christian, specify sect: ____________________

Church of Christ Scientist

Evangelical

Church of Scientology

Episcopalian

Greek or Russian Orthodox

Hindu

Islam

Jewish

La Regla Lukumi

Lutheran

Methodist

Metropolitan Community Church (MCC)

Mormon

Muslim

Nation of Islam

Native American Church/Peyote church

New Age

Other Yoruba influenced faiths, specify: ____________________

Pagan

Protestant

Santeria

Shango

Spiritual

Tribal specific religious system, specify: ____________________

Umbanda

Unitarian Universalist

Voodoo/Vonduh/Vundun

Gay religious group

Wicca

12-step

Other, specify: ____________________

None

Don’t know

Refuse to answer

**Religiosity**

1. **What religion were you raised with? Please circle all that apply.**

Agnostic

Atheist

Baptist

Buddhist

Candomble

Catholic

Christian, specify sect: ____________________

Church of Christ Scientist

Evangelical

Church of Scientology

Episcopalian

Greek or Russian Orthodox

Hindu

Islam

Jewish

La Regla Lukumi

Lutheran

Methodist

Metropolitan Community Church (MCC)

Mormon

Muslim

Nation of Islam

Native American Church/Peyote church

New Age

Other Yoruba influenced faiths, specify: ____________________

Pagan

Protestant

Santeria

Shango

Spiritual

Tribal specific religious system, specify: ____________________

Umbanda

Unitarian Universalist

Voodoo/Vonduh/Vundun

Gay religious group

Wicca12-step

Other, specify: ____________________

None

Don’t know

Refuse to answer

**Religiosity**

1. **How religious do you consider yourself to be?**

Not at all religious
Not very religious

Somewhat religious

Very religious

I DON’T KNOW

RF

**Religiosity**

1. **How important is your religion to you?**

Not important at all

Not that important

Somewhat important

Very important

I DON’T KNOW

RF

**Spirituality**

1. **How spiritual do you consider yourself to be?**

Very spiritual

Somewhat spiritual

Not very spiritual

Not at all spiritual

I DON’T KNOW

RF

**Spirituality**

1. **How important is your spirituality to you?**

Very important

Somewhat important

Not that important

Not important at all

I DON’T KNOW

RF

**Section C. Healthcare Services Usage/Engagement**

***This section is INTERVIEWER-ADMINISTERED***

For this next section, I’ll be asking you about general health questions.

**ALL participants**

**General Healthcare**

1. **What is your weight?**

Pounds: __________

I DON’T KNOW

RF

1. **What is your height?**

____ feet ____ inches

1. **In general, would you say your health is:**

Poor
Fair
Excellent

Good

Very good

I don’t know

Refuse

1. **Is there something about your health that you are worried about?**

No

Yes, specify:

I DON’T KNOW
RF

1. **In general, how happy have you been with your personal life during the past 12 months?**

Not happy at all

Not very happy

Fairly happy

Extremely happy

I DON’T KNOW

RF

1. **How many hours per night do you usually sleep?**

Hours: ____________

I DON’T KNOW

RF

1. **Do you feel like you sleep…**

Not enough hours

The right number of hours

Too many hours

I DON’T KNOW
RF

1. **In the past 7 days, how many days did you do exercise or participate in physical activity**

**for at least 20 minutes? The activity should have made you sweat and breathe hard, such**

**as basketball, soccer, running, swimming laps, fast bicycling, fast dancing, or similar aerobic**

**activities.**

0 days

1 day

2 days

3 days

4 days

5 days

6 days

7 days

I DON’T KNOW

RF

1. **During the past 7 days how many times did you eat fruit? (Do not count fruit juice.)**

I did not eat fruit during the past 7 days

1 to 3 times during the past 7 days

4 to 6 times during the past 7 days

1 time per day

2 times per day

3 times per day

4 or more times per day

I DON’T KNOW

RF

1. **How many times did you eat a green salad?**

I did not eat green salad during the past 7 days

1 to 3 times during the past 7 days

4 to 6 times during the past 7 days

1 time per day

2 times per day

3 times per day

4 or more times per day

I DON’T KNOW

RF

1. **Is there any place you go to if you are sick or need advice about your health?**

**(CARD 9)**

Acupuncturist

Botanica

Community health center (or public health clinic)

Company or work-affiliated clinic

Doctor’s office (group practice, clinic, HMO)

Herbalist or yerbero

Holistic healer

Hospital emergency room

Hospital outpatient clinic

Internet

Pastor or clergy person or priest

School clinic

Shaman

Spiritual healer or curandero

Other: _____________________

None

I DON’T KNOW

**Healthcare Utilization and Health Insurance**

These next set of questions are about health insurance.

1. **Do you have health insurance? (Choose all that apply).**

Insurance from work or school

Insurance under your parents

Insurance under your spouse or partner

Medical/Medicaid/My Health LA

Other insurance coverage: ____________

No insurance coverage

I DON’T KNOW

RF

1. **In the past 6 months, have you changed your health insurance?**

No Yes I don’t know

**SKIP Q.C14, IF RESPONSE TO Q.C12 IS “NO INSURANCE COVERAGE”**

1. **In the past 6 months, was there a period of time when you did not have any insurance?**

No Yes I don’t know

1. **If yes, why? (Check all that apply).**

Didn’t know how to sign up

I am not eligible for health insurance

Waited too long to sign up

Didn’t think I needed it

Other (please specify): __________________

1. **Do you have a place to go for healthcare (not including the clinic you are at today)?**

No Yes

1. **If yes, what is the name of the clinic(s) you go to?** ______________________

**SKIP Q.C18-C19, IF RESPONSE TO Q.C16 IS “NO”**

1. **Do you have a clinical/primary care provider?**

No Yes I don’t know

1. **How did you select your clinical/primary care provider? Choose all that apply.**

The insurance company selected it for me

Close to home

I use my family’s primary care provider

I looked up providers online

Friend’s referral

Other (please specify): __________________

1. **Did you see a doctor in the last 12 months?**

Yes No

1. **If no, what was the reason why? Select all that apply.**

I was not sick

I didn’t want to go
 I didn’t feel welcome
 Transportation issues
 I couldn’t get time off from work or school

No insurance

Other: _______________

1. **In the past 12 months, have you made and then missed an appointment with this provider?**

No Yes

1. **If yes, how many times did this happen in the past 12 months?**

_______ times

1. **If yes, why did you miss your appointment(s)? (Check all that apply.)** Did not have transportation

I was not sick

I had a conflict with work/school
I didn’t like the provider
I don’t feel welcome
Their process is too complicated
I had a bad experience with the doctor or at the clinic
Running late and wasn’t seen
The time was not good for me

I did not want to go

I had family issues

I forgot

I did not want a physical exam

Fear of mistreatment based on my gender identity

Anxiety related to past medical appointments

Depression

Other (please specify): ___________________

**ALL participants**

1. **What type of sexual and reproductive health issues does your primary provider raise with you? (Check all that apply.) (CARD 13a)**

Asks about whether I am sexual active

Asks about the gender of my sexual partners

Asks about the kind of sex that I am having

Asks about my sexual history

Tests for HIV

Tests for sexually transmitted infections

Discusses prevention of HIV and sexually transmitted infections

Discusses my reproductive health and family planning needs

Asks whether I am experiencing abuse in my relationship

Asks whether I have experienced sexual violence

Other (please specify): __________________________

My doctor does not discuss sexual or reproductive health issues

1. **Have you talked to your provider about your gender identity (i.e., gender nonconforming, transgender)?**

No Yes

1. **Has your doctor asked about your sexual orientation (i.e., gay, pansexual)?**

No Yes

1. **Has your doctor asked about the kind of sex you are having?**

No Yes

1. **In general, how satisfied are you with your primary care provider?**

Very dissatisfied

Somewhat dissatisfied

Somewhat satisfied

Very satisfied

1. **In the past 12 months, have you changed your primary provider?**

No Yes

1. **If yes, please tell us the reason(s) for changing your primary provider? (Check all that apply.)**

I didn’t like the provider

I needed a provider to treat an illness that I have

Provider did not feel comfortable managing my illness

Too far away

Didn’t like the feel of the office/clinic

Other (please specify): __________________________

1. **Have you ever been diagnosed with any of the following chronic illnesses? (Check all that apply.) (CARD 10)**

AIDS
Irritable bowel syndrome

Arthritis
Herpes

Asthma

Cancer

Chronic fatigue syndrome

Chronic Kidney Disease
Anxiety
Bipolar disorder
Schizophrenia
Attention deficit disorder
Obsessive compulsive

Diabetes

Fibromyalgia
Lyme diseases

Heart disease

Hepatitis

HIV

HPV (Human Papillomavirus)

Hypertension
Crohn’s disease
Insomnia
Post-traumatic stress
Depression

Other: _______________

Panic disorder
Drug or alcohol dependence

Peptic Ulcer
Eating disorders

Sickle cell disease

I have not been diagnosed with a chronic illness

**HIV+ only and those with other chronic illnesses**

1. **Are you currently taking medication for any of these illnesses?**

No Yes

***For these next few questions, please only think about the medication(s) you are taking for your chronic illness. THIS DOES NOT INCLUDE MEDICATIONS FOR HIV OR AIDS****.*

**Participants with chronic illnesses (NOT INCLUDING RESPONDENTS WHO ONLY HAVE HIV OR AIDS)**

**SKIP Q.C34-C43, IF RESPONSE TO Q.C32 IS “HIV” OR “AIDS” ONLY**

**SKIP Q.C34-C43, IF RESPONSE TO Q.C33 IS “NO”**

1. **In the past 12 months, was there any time when you weren’t able to get your medications?**

No Yes

1. **If yes, why not? (Check all that apply)**

Cost was too much

No insurance

Health plan problem

Can’t find pharmacy that accepts my insurance

Not available in area/transport problems

Not convenient times/could not get to the pharmacy

Did not know where to go

Refused to go

Lack of resources at school

Forgot

Other____________________________________________________

**Medication adherence**

The next section will be about medication habits.

1. **Do you sometimes forget to take your medicine?** No Yes
2. **People sometimes miss taking their medicines for reasons other than forgetting. Thinking over the past 2 weeks, were there any days when you did not take your medicine?** No Yes
3. **Have you ever cut back or stopped taking your medicine without telling your doctor because you felt worse when you took it?** No Yes
4. **When you travel or leave home, do you sometimes forget to bring along your medicine?** No Yes
5. **Did you take all your medicines yesterday?** No Yes
6. **When you feel like your symptoms are under control, do you sometimes stop taking your medicine?** No Yes
7. **Taking medicine every day is a real inconvenience for some people. Do you ever feel hassled about sticking to your treatment plan?** No Yes
8. **How often do you have difficulty remembering to take all your medicine?**

Never/rarely

Once in a while

Sometimes

Usually

All the time

**HIV+ participants ONLY**

**ART adherence**

Now we’re going to ask you some questions about your HIV medications.

1. **Are you currently taking pills or other medicines to treat your HIV?**

No

Yes

“Many people with HIV have many pills or other medicines to take at different times during the day. Often people find it hard to always remember to take their pills or medicines Sometimes people get busy and forget to carry their pills with them. Other times people find it hard to remember to take their pills like their doctor told them to, such as “with food” or “on an empty stomach” or “every 8 hours.” Other times people decide to skip pills to avoid side effects (like feeling sick to your stomach) or to just not take pills that day.

It is important for us to understand what people with HIV are really doing with their pills or medicines. Please tell us what you are actually doing. Don’t worry about telling us you don’t take all your pills or medicines. We want to know what is really happening, not what you think we want to hear.

The following questions ask you about the dose of pills that are prescribed for you. By “dose of pills,” we mean the quantity of pills or medicines prescribed to be taken at one particular time (for example, 3 pills before bedtime).”

1. **“How many times during the day has your doctor told you to take a dose of medicine (pills or other medicines) to treat your HIV?”**

Once a day
 Twice a day

Three times a day

Four or more times a day

I don’t know

1. **“What is the total number of pills your doctor has told you to take each day?”**

|_____|_____| Pills

Now we’re going to ask you to think about last weekend (Friday, Saturday and Sunday). Sometimes taking medication can be even more difficult on weekends.

1. **Thinking about the last weekend, how many doses did you miss? (Please type in a number from 0 (which means you didn’t miss any doses last weekend) to as many doses as you might have missed).**

|_____|_____| Doses

*******Note to Billy:*** *Allow responses from 0 to 12. If response is greater than 12, please display, “You have entered an invalid response. Please re-enter.”*

For answers greater than 9, “I want to confirm the number of doses of pills that you missed taking last weekend. The number that you entered is displayed on the screen. Is that correct?”

1. **Around how many times did you miss taking a dose of pills in the past 7 days? Please type in a number from 0 (which means you didn’t miss any doses of pills) to as many times as you might have missed taking a dose of pills.”**

|_____|_____| Times

1. **Did anything happen in the last 7 days that made it more difficult to take the medicine your doctor prescribed for you?”**

Yes
 No

**IF Q.C47 OR Q.C48 > 0, ASK Q.C50. OTHERWISE, GO TO Q.C51**

1. **If you missed taking any pills over the last 7 days, what were some of the reasons? [Check all that apply] (CARD 11)**

Can’t get pill at drug store

Ran out of prescription

Did not have health insurance to pay for the prescriptions

Made me sick to my stomach/tasted bad

Forgot

I got a headache or rash or other physical symptom

It got in the way of my daily schedule (school, work)

Didn’t feel like taking it, needed a break

Change in living situation, moved

Worried that someone would find out about the HIV

Got sick with another illness, wasn’t feeling well (e.g., cold, flu, etc.)

Don’t think I need the pills anymore, I can stay healthy without them

Family and/or friends don’t help me remember

Family and/or friends tell me I shouldn’t take them

Nowhere to keep the pills at school or work

Don’t understand why I have to take the pills

I keep getting sick even when I do take the pills

Taking it reminds me of the HIV. I just want to forget about the diagnosis

Other, specify ___________________________

I don’t know

1. **In the last 7 days, did you do anything to help you remember to take your pills (or medicine)?**

No

Yes

**SKIP Q.C52, IF RESPONSE TO Q.C51 IS ‘NO’**

1. **Please select the things you did to help you remember to take your doses of pills. Choose
    all that apply. (CARD 12)**

Text Message

Alarm on cell phone

Internet reminder system

Labels

Calendars

Pill boxes

Beepers

Monitoring caps (MEMS)

Timers

Programmable wrist watches

Diary

Buddy system (someone who helps me to remember)

Always taking the pills when a certain thing happens during the day (for instance, every time you eat breakfast)

Other, specify ________________________

I don’t know

1. **Has your doctor offered or prescribed pills or other medicines for your HIV infection but you never started taking those medicines?**

No

Yes

1. **Have you been prescribed pills or other medicines for your HIV infection that you took for a while but have now stopped completely?**

No

Yes

1. **What were some of the reasons that you stopped taking pills or other medications for your HIV infection? [Check all that apply] (CARD 11)**

Couldn’t get pill at drug store

Ran out of prescription and never started again

Did not have health insurance to pay for the prescriptions

Made me sick to my stomach/tasted bad

Forgot

I got a headache or rash or other physical symptom

It got in the way of my daily schedule (school, work)

Didn’t feel like taking it, needed a break

Change in living situation, moved

Worried that someone would find out about the HIV

Got sick with another illness, wasn’t feeling well (e.g., cold, flu, etc.)

Don’t think I needed the pills anymore; I can stay healthy without them

Family and/or friends didn’t help me remember

Family and/or friends told me I shouldn’t take them

Nowhere to keep the pills at school or work

Didn’t understand why I had to take the pills

I kept getting sick even when I did take the pills

Taking it reminded me of the HIV; I just wanted to forget about the diagnosis

I was afraid it might have negative reaction with my hormone therapy

Other, specify ________________________

**Please tell me if you Disagree, are Not Sure, or Agree with these 5 statements.**

1. **I don't take my medicines when they make me feel bad.**
2. **I don't take my medicines when I am too tired.**
3. **I don't take my medicines when I am feeling down or low.**
4. **I don't take my medicines because it tastes bad.**
5. **I don't take my medicines when I feel good.**

**HIV+ only and those with other chronic illnesses**

1. **How satisfied are you with the way in which your primary care provider communicates with you [about your illness? Or in general]?**

Very dissatisfied

Somewhat dissatisfied

Somewhat satisfied

Very satisfied

**HIV+ only and those with other chronic illnesses**

1. **How confident are you that your primary provider/primary provider team understands your illness(es)/condition(s)?**

Not confident at all

Not very confident

Somewhat confident

Very confident

**HIV+ only**

1. **What type of sexual and reproductive health issues does your primary provider raise with you? (Check all that apply.) (CARD 13b)**

Discusses how my HIV medication might affect my reproductive health

Discusses how my HIV status might affect family planning

Discusses how my HIV status might affect sexual activity

Other (please specify): __________________________

My doctor does not discuss sexual or reproductive health issues

**SKIP Q.C64-C73, IF ANSWER TO Q.C64 IS “I HAVE NOT BEEN DIAGNOSED WITH A CHRONIC ILLNESS”**

1. **Do you have a specialty provider?**

No Yes My specialty provider is also my primary care provider

**SKIP Q.C65-C73, IF ANSWER TO Q.C64 IS “NO”**

**SKIP Q.C65, IF RESPONSE TO Q.C64 IS “MY SPECIALTY PROVIDER IS ALSO MY PRIMARY CARE PROVIDER”**

1. **What is the name of the clinic(s) you go to for your specialty provider?**

_________________________

1. **What is your provider’s specialty (e.g., cardiology, pulmonology, nephrology)?**

_________________________

**SKIP Q.C67, IF RESPONSE TO Q.C64 IS “MY SPECIALTY PROVIDER IS ALSO MY PRIMARY CARE PROVIDER”**

1. **How did you select this provider?**

My insurance company/MediCal helped me select this provider

My primary provider referred me

It’s close to home

Other (please specify): _________________________

1. **In the past 6 months, how many times have you seen this provider?**

_______ times

I have not seen this provider yet

**SKIP Q.C69-C71, IF RESPONSE TO Q.C64 IS “MY SPECIALTY PROVIDER IS ALSO MY PRIMARY CARE PROVIDER”**

1. **In general, how satisfied are you with your specialty provider?**

Very dissatisfied

Somewhat dissatisfied

Somewhat satisfied

Very satisfied

1. **How confident are you that your specialty provider understands your illness?**

Not confident at all
Not very confident

Somewhat confident

Very confident

I don’t know

1. **In the past 12 months, to your knowledge, has your specialty provider communicated with your primary care provider about your illness and history?**

No Yes

1. **Do you have other specialists?**

No Yes

**SKIP Q.C73, IF RESPONSE TO Q.C72 IS “NO”**

1. **If yes, how many?** ________________________________

**Barriers to Care**

1. **How likely are you to see a primary care provider for routine care?**

Very unlikely

Unlikely

Somewhat likely

Likely

Very likely

**SKIP Q.C75, IF RESPONSE TO Q.C74 IS “VERY LIKELY” OR “LIKELY”**

1. **What are some things the make it hard for you to see a primary care provider for routine care? (Check all that apply)**

Anxiety about going to the doctor

Past negative experience with a provider

No health insurance

Cannot afford copay

Depression

Distance/transportation issues

Difficulty understanding how or where to go

Difficult to get an appointment

Difficult to get an appointment that fits my schedule

Healthcare is not a priority for me right now

Do not want a physical exam

Fear of mistreatment based on my gender identity

Fear of mistreatment based on my race/ethnicity

Fear or mistreatment based on my body size/weight

Fear of being diagnosed with HIV

Fear of being diagnosed with another health condition

I am healthy and do not need to go to the doctor

1. **How important is it for you to have a primary care provider who specializes in working with transgender or gender nonconforming people?**

Very unimportant

Somewhat unimportant

Neither important nor unimportant

Somewhat important

Very important

1. **How important is it for you to receive medical care of any kind at an LGBT-centered clinic?**

Very unimportant

Somewhat unimportant

Neither important nor unimportant

Somewhat important

Very important

1. **Does seeing a provider who specializes in transgender health make you feel more or less comfortable in the healthcare setting?**

Much less comfortable

Somewhat less comfortable

Does not make a difference

Somewhat more comfortable

Much more comfortable

1. **How likely is it that you would make future changes to your diet or exercise routine if recommended to do so by your care provider?**

Very unlikely

Unlikely

Somewhat likely

Likely

Very likely

**Hospitalizations**

1. **In the past 12 months, have you been hospitalized? *(Note to INTERVIEWER: Please
    explain that “hospitalized” refers to being admitted to a hospital for treatment or
    monitoring)*.**

Yes No

1. **If yes, how many times were you hospitalized?**

_______ times

1. **If yes, can you tell me the number of nights you have been in the hospital in the past 12 months?**

_______ nights

1. **If yes, thinking about your last hospitalization, please tell us the reason for hospitalization.**

________________________________________

1. **In the past 12 months, have you visited an emergency room?**

No Yes

1. **If yes, how many times have visited an emergency room?**

_______ times

1. **If yes, thinking about your last visit to the emergency room, please tell us the reason for the visit.**

________________________________________

1. **If yes, why did you visit the emergency room and not your regular doctor?**

I don’t have a regular doctor

It takes too long to get an appointment with my regular doctor

This is where I go whenever I have a health problem

Problem was too urgent

My doctor advised me to visit the emergency room

Other: ­­­­____________________

I don’t know

**ALL participants**

1. **Have you ever seen a mental health care provider in order to get a letter for gender affirming health care (e.g., hormones)?**

No

Yes

Don’t Know

1. **In the past 12 months, was there any time you have needed or wanted mental health care or counseling for other reasons?**

No

Yes

Don’t Know

**SKIP Q.C90, IF RESPONSE TO Q.C89 IS “NO”**

1. **What did you need or want mental health care or counseling for? (If you needed mental health care or counseling more than once in the last 12 months please check what you needed/wanted care for on your 3 most recent visits).** **Choose all that apply.
    (CARD 14)**

Anger

Anxiety

Confusion

Difficulties with concentration or attention

Distress in social situations

Drug or alcohol use

Excessive fear and worry

Family conflict

Gender dysphoria or stress related to your gender identity

Guilt

Hallucinations

Helplessness

Hopelessness

Impaired memory and concentration

Indecisiveness and confusion

Irritability

Lack of emotional responsiveness

Lack of energy

Lack of inhibitions

Loss of motivation

Mind racing or going blank

Mood swings

Neglect of responsibilities

Obsessive or compulsive behavior

Overeating or loss of appetite

Pessimism

Phobic behavior

Post-traumatic stress

Psychosis

Reduced energy and motivation

Relationship problems

Restlessness

Sadness

Self-blame

Self-criticism

Self-harm

Sleep disturbance

Social isolation or withdrawal

Thoughts of death and suicide

Weight loss or gain

Withdrawal from others

Other: _______________________

1. **Did you receive all the mental health care or counseling you needed or wanted?**

No

Yes

1. **If you did not receive all the mental health care or counseling you needed or wanted, why not? (CARD 15)**

Cost was too much

No insurance

A health plan problem

I can’t find provider who accepts my insurance

Services not available in area or transport problems

No convenient times/could not get appointment

The provider did not know how to treat or provide care

The provider did not know anything about working with transgender, gender non-conforming or non-binary people

Dissatisfaction with provider

I did not know where to go for treatment

I was afraid of what people would think of me

I refused to go

Treatment is ongoing

No referral

Lack of resources at school

I did not go to the appointment, neglected the appointment, forgot the appointment, or never made the appointment

Other: ______________________________

**ALTERNATIVE HEALTH CARE**The next few questions are about Alternative Health Care.

Mainstream medicine often involves going to a doctor, taking medication, or having surgery. Alternative healthcare and treatments include healthcare approaches that are **outside of mainstream medicine**. Some of the most common alternative healthcare approaches are yoga, meditation, and special diets.

**SKIP Q.C93-C96, IF RESPONSE TO Q.C32 IS “I HAVE NOT BEEN DIAGNOSED WITH A CHRONIC ILLNESS”**

**HIV+ only and those with other chronic illnesses**

1. **During the past 12 months did you use any type of alternative health care or treatment?**

No

Yes

Don’t know

1. **If yes, what type of alternative healthcare or treatment did you use? (Check all that apply.)**

**(CARD 16)**

Medicinal cannabis/marijuana

Acupuncture

Chiropractic care

Relaxation therapies

Herbal supplements

Deep breathing exercises

Yoga

Chelation therapy

Energy healing therapy

Hypnosis

Massage

Naturopathy

Traditional healers

Other, please specify: ____________________

Don’t know

**SKIP Q.C95-C96, IF ANSWER TO Q.C94 IS *NOT* “MEDICAL MARIJUANA”**

1. **How effective do you feel that marijuana has been in treating your health condition in the last 6 months (including symptoms of condition and/or side effects of medication)?**

Not effective at all (1)

A little effective (2)

Somewhat effective (3)

Quite effective (4)

Very effective (5)

1. **[IF ANSWER TO Q.C95 IS 2 OR MORE] What are the top are the top three symptoms, medication side effects, or other health issues that marijuana has been effective in treating?**
2. ________________________
3. ________________________
4. ________________________

**Hormones and Silicone Use**

The following questions are about any past use of silicone injections or hormones.

1. **Were you ever prescribed hormone blockers, before you were age 12? This would slow down the onset of puberty?**

No Yes I don’t know

1. **Have you ever taken gender affirming hormone therapies? This would include estradiol, anti-androgens or testosterone?**

No Yes I don’t know

**SKIP Q.C99, IF ANSWER TO Q.C98 IS “NO”**

1. **Did you use these hormones in the past 12 months?**

No Yes I don’t know

1. **Do you currently have a prescription for hormones under the supervision of a healthcare provider?**

No Yes I don’t know

**SKIP Q.C101, IF ANSWER TO Q.C100 IS “YES”**

1. **How are you typically accessing hormones? (Choose all that apply)**

Off the Internet

From someone selling it

From a friend

Other, please specify: __________________

I don’t know

**SKIP Q.C102-C103, IF ANSWER TO Q.C98 IS “NO”**

1. **How do you typically take hormones?**

Take orally (if testosterone, this is not applicable)

Intermuscular injection

Subcutaneous injection

Patch or gel

Other, please specify: __________________

1. **Have you ever injected or been injected with hormones?**

No Yes I don’t know

**SKIP Q.C104-C108, IF ANSWER TO Q.C103 IS “NO”**

1. **In what year was your last hormone injection? ______________**
2. **In what month was your last hormone injection? ______________**

1. **How often did you get hormone injections in the past 12 months?**

Less than once a month

Once a month

More than once a month (or twice a week)

I have not injected in the past 12 months

I don’t know

**SKIP Q.C107-C108, IF ANSWER TO Q.C106 IS “I HAVE NOT INJECTED IN THE PAST 12 MONTHS” OR “I DON’T KNOW”**

1. **How are you getting hormones for your injections? Check all that apply.**

Through a prescription from a doctor

Off the Internet

From someone selling it

From a friend

Other, please specify: __________________

I don’t know

1. **In the last 12 months, who performed the hormone injections? Check all that apply.**

Myself

Doctor or nurse in the US

Doctor or nurse in another country

A person who is not a doctor or nurse but regularly performs this service for transgender people

A friend

Other, please specify: __________________

I don’t know

***The next questions are about substances like silicone that are injected to change the shape of the body.***

1. **Have you ever injected, or been injected with, a substance like silicone to change the shape of your body?**

No Yes I don’t know

**SKIP Q.C110-C114, IF ANSWER TO Q.C109 IS “NO”**

1. **What substances did you inject to change the shape of your body?**

Silicone

Some other substance (please, specify: __________________)

Silicone and some other substance (please, specify: __________________)

I don’t know

1. **What was the date of your most recent silicone injection?** __________________
2. **In the past 12 months, how often were you injected with silicone or some other substance to change your appearance?**

Never

Once

Twice

3 to 5 times

6 to 10 times

More than 10 times

I don’t know

1. **Where did you get silicone or other substance you injected into your body?**

Through a prescription from a doctor

From someone selling it

From a friend

Other, please specify: __________________

I don’t know

1. **In the last 12 months, who performed the injections?**

Myself

Doctor or nurse in the US

Doctor or nurse in another country

A person who is not a doctor or nurse but regularly performs this service for transgender people

A friend

Other, please specify: __________________

I don’t know

**Health-related quality of life**

**Participants with chronic illnesses (including HIV and AIDS) ONLY**

1. **Compared to other people your age, would you say your health is:**

Poor
 Fair

Good

Excellent

**Trust/mistrust of healthcare system (CARD 17)**

The following questions are about the healthcare system. Please let me know if you Strongly Disagree, Disagree, are Not Sure, Agree, or Strongly Agree with the following statements.

**ALL participants**

1. **Medical experiments can be done on me without my knowing about it.**

Strongly disagree

Disagree

Not sure

Agree

Strongly agree

1. **My medical records are kept private.**

Strongly disagree

Disagree

Not sure

Agree

Strongly agree

1. **People die every day because of mistakes by the health care system.**

Strongly disagree

Disagree

Not sure

Agree

Strongly agree

1. **When they take my blood, they do tests they don’t tell me about.**

Strongly disagree

Disagree

Not sure

Agree

Strongly agree

1. **If a mistake were made in my health care, the health care system would try to hide it from me.**

Strongly disagree

Disagree

Not sure

Agree

Strongly agree

1. **People can get access to my medical records without my approval.**

Strongly disagree

Disagree

Not sure

Agree

Strongly agree

1. **The health care system cares more about holding costs down than it does about doing what is needed for my health.**

Strongly disagree

Disagree

Not sure

Agree

Strongly agree

1. **I receive high-quality medical care from the health care system.**

Strongly disagree

Disagree

Not sure

Agree

Strongly agree

1. **The health care system puts my medical needs above all other considerations when treating my medical problems.**

Strongly disagree

Disagree

Not sure

Agree

Strongly agree

1. **Some medicines have things in them that they don’t tell you about.**

Strongly disagree

Disagree

Not sure

Agree

Strongly agree

You mentioned earlier that you have been diagnosed with a chronic illness or condition. These next set of questions will ask you about your experiences. Please use the following scale: Never, Very Seldom, Often, and All the Time.

|  | **Health-related quality of life (CARD 18)** Include instructions to reference their selected chronic illness/disease. | **Never** | **Very Seldom** | **Sometimes** | **Often** | **All the Time** |
| --- | --- | --- | --- | --- | --- | --- |
|  | How often are you embarrassed by having to deal with your chronic illness/condition in public? |  |  |  |  |  |
| 1. 1 | How often do you feel physically ill? |  |  |  |  |  |
|  | How often does your chronic illness/condition interfere with your family life? |  |  |  |  |  |
|  | How often do you have a bad night’s sleep? |  |  |  |  |  |
|  | How often do you find your chronic illness/condition limiting your social relationships and friendships? |  |  |  |  |  |
|  | How often does your chronic illness/condition interfere with your sex life? |  |  |  |  |  |
|  | How often do you miss work, school, or household duties because of your chronic illness/condition? |  |  |  |  |  |
|  | How often do you tell others about your chronic illness/condition? |  |  |  |  |  |
|  | How often are you teased because you have your chronic illness/condition? |  |  |  |  |  |
|  | How often do you find that your chronic illness/condition prevents you from participating in school activities (for example, being active in a school play, being on a sports team, being in a school band, etc.)? |  |  |  |  |  |
|  | How often do you feel that your chronic illness/condition is limiting your career or what you will be able to do in the future? |  |  |  |  |  |
|  | How often do you feel that your parents worry too much about your chronic illness/condition? |  |  |  |  |  |
|  | How often do you find that close family members (for example, brothers, sisters, cousins) tease you about your chronic illness/condition? |  |  |  |  |  |

|  | **(CARD 19)** | **Never** | **Very Seldom** | **Sometimes** | **All the Time** | **Does Not Apply** |
| --- | --- | --- | --- | --- | --- | --- |
|  | How often do you worry about whether you will get married? |  |  |  |  |  |
|  | How often do you worry about whether you will have children? |  |  |  |  |  |
|  | How often do you worry about whether you will not get a job you want? |  |  |  |  |  |
|  | How often do you worry about whether you will be able to complete your education? |  |  |  |  |  |
|  | How often do you worry about whether you will miss school or work? |  |  |  |  |  |
|  | How often do you worry about whether you will be able to take a vacation or a trip? |  |  |  |  |  |
|  | How often do you worry that your body looks different because of your chronic illness/condition? |  |  |  |  |  |
|  | How often do you worry that because of your chronic illness/condition, you are behind in terms of dating, going to parties, and keeping up with your friends? |  |  |  |  |  |

**Section D. HIV & STI Testing/Status**

***This section is SELF-ADMINISTERED***

For this next section, you'll be using the laptop to answer the questions. These questions will be about testing behaviors for STDs, including HIV.

**Tested for STI**

1. **Have you ever been tested for sexually transmitted disease or infection (STD/STI)?**

No

Yes

I’m not sure

Refuse to answer

**SKIP Q.D2-D3, IF Q. D1 IS “NO” OR “I’M NOT SURE”**

**STI Diagnosis**

1. **How many times have you been told by a health care provider or doctor that you have a**

**sexually transmitted disease or infection (STD/STI?)**

*Answer Scale:* Never, 1 time, 2 times, 3 or more times, Don't know, Refuse to answer

Gonorrhea _____________________

Syphilis _____________________

Chlamydia _____________________

Herpes _____________________

HPV / Genital warts _____________________

Hepatitis A / B _____________________

Hepatitis C _____________________

Scabies /crabs _____________________

Trichomoniasis _____________________

Bacterial Vaginosis _____________________

Other _____________________

**STI Treatment**

1. **Where did you go for treatment? (Check all that apply)**

Holistic / alternative healer

Community / public health clinic

School or work clinic

Doctor’s office

Hospital ER

Hospital clinic

Internet

Spiritual or religious leader

Did not receive treatment

**Last HIV test**

1. **When was the LAST time you were tested for HIV?**

I have never been tested for HIV

Less than 3 months ago

Between 3 and 6 months ago

More than 6 months to 1 year ago

More than 1 year ago

I don't know

Refuse to answer

**SKIP Q.D5, IF RESPONSE TO Q.D4 IS “LESS THAN 3 MONTHS AGO” OR “BETWEEN 3 MONTHS AND 6 MONTHS AGO”**

**Reasons for not testing**

1. **If you have not tested for HIV within the last six months, why have you not been tested for HIV? (Check all that apply.)**

I don't think I am at risk for HIV

I don't know where to get tested

It’s hard to access places where testing is done

I feel embarrassed to be seen in a HIV testing facility

I'm too scared to get tested

Other: ­­­­­­­­­­­­­________________________

I am HIV positive

I don't know

Refuse to answer

**SKIP Q.D6-D8, IF RESPONSE TO Q.D4 IS “I HAVE NEVER BEEN TESTED FOR HIV”**

**SKIP Q.D6-D9, IF RESPONSE TO Q.D5 IS “I AM HIV POSITIVE”**

**HIV test location**

1. **Where did you get tested?**

Rapid testing in a van

Rapid testing at a clinic

At a clinic (NOT rapid testing)

At a doctor's office

I don't know

Refuse to answer

**SKIP Q.D7-D8, IF RESPONSE TO Q.D5 IS “I AM HIV POSITIVE”**

**HIV results**

1. **Did you get your test results?**

No

Yes

I don't know

Refuse to answer

**HIV Status**

1. **What was the result of your last HIV test?**

Negative

Positive

I don't know

Refuse to answer

**SKIP Q.D9, IF RESPONSE TO Q.D5 IS “I AM POSITIVE”**

**SKIP Q.D9, IF RESPONSE TO Q.D8 IS “POSITIVE”**

1. **How likely do you think you will get HIV?**

Not likely

Somewhat likely

Likely

Very likely

**HIV+ participants only**

**Age at HIV+**

1. **How old were you when you first tested positive for HIV?** _____________________

**ALL participants**

1. **Have you been diagnosed with AIDS?**

No

Yes

**Know HIV+/AIDS**

1. **Do you know anyone who has HIV?**

Yes

No

I don't know

Refuse to answer

**Know HIV+/AIDS**

1. **Do you know anyone who has AIDS?**

Yes

No

I don't know

Refuse to answer

**HIV+ participants ONLY**

**HIV Stigma**

This set of questions asks about some of your experiences, feelings, and opinions as to how people with HIV feel and how they are treated. Please do your best to answer each question.

For each item, circle your answer: Strongly disagree (SD), disagree (D), agree (A), or strongly agree (SA).

1. **Having HIV makes me feel unclean**....................................... SD D A SA
2. **Most people think that a person with HIV is disgusting**............... SD D A SA
3. **Having HIV makes me feel that I'm a bad person**.................. SD D A SA
4. **Most people with HIV are rejected when others find out**......................................... SD D A SA
5. **I am very careful who I tell that I have HIV**.......................... SD D A SA
6. **I have been hurt by how people reacted to learning I have HIV**............................. SD D A SA
7. **I worry that people who know I have HIV will tell others**.................................... SD D A SA
8. **I have lost friends by telling them I have HIV**....................... SD D A SA
9. **I have stopped socializing with some people because of their reactions of my having HIV**............... SD D A SA
10. **I feel that I am not as good a person as others because I have HIV**............... SD D A SA

**General HIV & Testing-Specific Knowledge**

**ALL participants**

1. **How would you rate your knowledge about HIV/AIDS in general?**

**1** - Very low **2** - Low **3** - Moderate **4** - High **5** - Very High

1. **How would you rate your knowledge specifically related to HIV testing?**

**1** - Very low **2** - Low **3** - Moderate **4** - High **5** - Very High

Please tell me if you Strongly Disagree, Disagree, Agree, or Strongly Agree with the next set of statements.

1. **New treatments for HIV have brought hope for a cure**

Strongly disagree

Disagree

Agree

Strongly agree

1. **HIV will soon be controllable like diabetes**

Strongly disagree

Disagree

Agree

Strongly agree

1. **My community is upbeat about stopping AIDS**

Strongly disagree

Disagree

Agree

Strongly agree

1. **There will be a cure for HIV in the next few years**

Strongly disagree

Disagree

Agree

Strongly agree

1. **People in my community are less worried about AIDS because of new treatments**

Strongly disagree

Disagree

Agree

Strongly agree

1. **My community has become relaxed about AIDS**

Strongly disagree

Disagree

Agree

Strongly agree

1. **New HIV treatments can be taken after unsafe sex to prevent HIV infection**

Strongly Agree

Agree

Disagree

Strongly Disagree

1. **HIV-positive people who take drug cocktails are less likely to infect sex partners during unsafe sex**

Strongly disagree

Disagree

Agree

Strongly agree

1. **It is safe to have sex without a condom if I have an undetectable viral load**

Strongly disagree

Disagree

Agree

Strongly agree

1. **New AIDS treatments make it easier to relax about unsafe sex**

Strongly disagree

Disagree

Agree

Strongly agree

1. **I practice more unsafe sex because of new HIV treatments**

Strongly disagree

Disagree

Agree

Strongly agree

1. **How well do you understand what a T-helper (CD4) cell count is and what it means?**

I have somewhat or no understanding

I have complete understanding

1. **Is the goal of treatment to make the CD4 count go up or down?**

Up Down

1. **How well do you understand what a viral load is and what it means?**

I have somewhat or no understanding

I have complete understanding

1. **Is the goal of treatment to make the viral load go up or down?**

Up Down

**HIV+ participants only**

1. **Do you know your most recent CD4 cell count?**

No

Yes, please specify: _____________________

1. **Do you know your most recent HIV viral load test results?**

No

Yes, please specify: _____________________

**PrEP Knowledge and Use**

The next couple of questions will be about your use of pre-exposure prophylaxis (PrEP) medications, such as Truvada, that can be used to reduce the risk of HIV infection by taking a pill.

1. **Before today, had you ever heard about PrEP?**

No

Yes

I don’t know

**SKIP Q.D44-D63, IF RESPONSE TO Q.D43 IS “NO” OR “I DON’T KNOW”**

1. **Have you ever received information about this medication?**

No

Yes

I don’t know

**SKIP Q.D45, IF RESPONSE TO Q.D44 IS “NO”**

1. **If yes, where did you get information on how to use PrEP?**

Pharmacist
 Doctor
 Friends
 HIV counselor

Social media, please specify: _______________
 Other: _______________

**SKIP Q.D46-D49, IF RESPONSE TO Q.C32 IS “HIV” OR “AIDS”**

1. **How likely would you be to take PrEP is it was offered to you for free?**

Would definitely not

Would probably not
Might

Would probably

Would definitely

1. **How likely would you be to take PrEP if your doctor recommended it?**

Would definitely not

Would probably not
Might

Would probably

Would definitely

1. **How likely would you be to take PrEP if an HIV testing counselor discussed it with you?**

Would definitely not

Would probably not
Might

Would probably

Would definitely

1. **To what extent do you think taking PrEP would influence your condom use?** Significantly less likely

Somewhat less likely
 Would not change

Somewhat more likely

Significantly more likely

1. **In your entire life, have you ever taken any pre-exposure prophylaxis (PrEP) medication, such as Truvada, to reduce your risk of HIV transmission?**

No Yes

1. **During the past 6 months when you were having anal or frontal sex, were you taking any pre-exposure prophylaxis (PrEP) medication, such as Truvada, to reduce your risk of HIV transmission?**

No Yes I have not had anal or frontal sex

**SKIP Q.D52, IF RESPONSE TO Q.D51 IS “NO” OR “I HAVE NOT HAD ANAL OR FRONTAL SEX”**

1. **Which of the following answer options best represents the frequency in which you have taken PrEP while having anal or frontal sex in the past 6 months?**

- I was using PrEP before I started having anal or frontal sex with my partner, and I **HAVE NOT** missed any recommended doses.
- I was using PrEP before I started having anal or frontal sex with my partner, but I **HAVE** missed some recommended doses.
- I started using PrEP after I already was having anal or frontal sex with my partner, and I **HAVE NOT** missed any recommended doses.
- I started using PrEP after I already was having anal or frontal sex with my partner, but I **HAVE** missed some recommended doses.

1. **During the past 6 months, was your sexual partner(s) taking any pre-exposure prophylaxis (PrEP) medication, such as Truvada, to reduce their risk of HIV transmission?**

No

Yes

I don’t know

**PrEP Medication Adherence**

**SKIP Q.D54, IF ANSWER TO Q.D51 IS “NO” OR “I HAVE NOT HAD ANAL OR FRONTAL SEX”**

1. **Were you prescribed PrEP or did you get it from a friend?**

I was prescribed PrEP

I got PrEP from a friend

I have not taken PrEP

**SKIP Q.D55-D62, IF RESPONSE TO Q.C32 IS “HIV” OR “AIDS”**

**SKIP Q.D55-D62, IF ANSWER TO Q.D43 IS “NO”**

1. **Are you currently taking any pre-exposure prophylaxis (PrEP) medication, such as Truvada, to reduce your risk of HIV transmission?**

No

Yes

**SKIP Q.D56-D62, IF ANSWER TO Q.D55 IS “NO”**

1. **What is the name of the PrEP medication that you are currently taking?**

__________________________________

1. **When did you first begin taking this medication?** **If you are having a difficult time
    remembering, please provide us with your best estimate.**

Month: __________________

Year: ____________________

1. **How often do you use PrEP?**

Every day

Only before having sex

Only if I know my partner’s HIV status

Other: _______________

1. **In the last 7 days, did you do anything to help you remember to take your PrEP medication?** No
    Yes

**SKIP Q.D60, IF RESPONSE TO Q.D59 IS ‘NO’**

1. **Please select the things you did to help you remember to take your PrEP medication.**

**[Check all that apply] (CARD 12)**

Text Message

Alarm on cell phone

Internet reminder system

Labels

Calendars

Pill boxes

Beepers

Monitoring caps (MEMS)

Timers

Programmable wrist watches

Diary

Buddy system (someone who helps me to remember)

Always taking the pills when a certain thing happens during the day (for instance, every time you eat breakfast)

Other, specify ________________________

I don’t know

1. **What has gotten in the way of you taking your regular PrEP doses in the last 30 days? [Check all that apply]**

Couldn’t get pill at drug store/pharmacy

Ran out of prescription and never started again

Did not have health insurance to pay for the prescriptions

Made me sick to my stomach/tasted bad

Forgot

I got a headache or rash or other physical symptom

It got in the way of my daily schedule (school, work)

Didn’t feel like taking it, needed a break

Change in living situation, moved

Worried that someone will think I have HIV

Got sick with another illness, wasn’t feeling well (e.g., cold, flu, etc.)

Don’t think I needed the pills anymore; I can stay healthy without them

Family and/or friends didn’t help me remember

Family and/or friends told me I shouldn’t take them

Nowhere to keep the pills at school or work

Didn’t understand why I had to take the pills

I kept getting sick even when I did take the pills

Taking it reminded me of HIV

Other, specify ________________________

I have not had any trouble taking my regular PrEP doses

1. **In the last 6 months, during a typical week (7 days), how many days did you normally use PrEP?**

0 times

1 time
 2 times

3 times

4 times

5 times

6 times

7 times

**SKIP Q.D63-D73, IF RESPONSE TO Q.D43 IS “NO” OR “I DON’T KNOW”**

**PrEP Stigma**

People have different opinions about PrEP. Please decide how much you agree with the following statements.

1. **I think people should take PrEP.**

Strongly disagree

Disagree

Neither agree nor disagree

Agree

Strongly agree

1. **I think condoms are a better choice than PrEP.**

Strongly disagree

Disagree

Neither agree nor disagree

Agree

Strongly agree

1. **People who are on PrEP sleep around.**

Strongly disagree

Disagree

Neither agree nor disagree

Agree

Strongly agree

1. **People who are on PrEP are irresponsible.**

Strongly disagree

Disagree

Neither agree nor disagree

Agree

Strongly agree

1. **Having sex with someone on PrEP is risky.**

Strongly disagree

Disagree

Neither agree nor disagree

Agree

Strongly agree

1. **People who are on PrEP are making a smart decision to protect their health.**

Strongly disagree

Disagree

Neither agree nor disagree

Agree

Strongly agree

1. **I would not trust someone who told me they were on PrEP.**

Strongly disagree

Disagree

Neither agree nor disagree

Agree

Strongly agree

1. **People who are on PrEP can’t control their sex drive.**

Strongly disagree

Disagree

Neither agree nor disagree

Agree

Strongly agree

1. **People who are on PrEP use it as an excuse to have sex without a condom.**

Strongly disagree

Disagree

Neither agree nor disagree

Agree

Strongly agree

1. **Many people on PrEP lie about whether or not they take it every day.**

Strongly disagree

Disagree

Neither agree nor disagree

Agree

Strongly agree

1. **People who are on PrEP are responsible.**

Strongly disagree

Disagree

Neither agree nor disagree

Agree

Strongly agree

**PEP Knowledge and Use**

PEP stands for post-exposure prophylaxis. PEP is an anti-HIV medication that you take after you think you may have been exposed to HIV. If taken correctly, PEP helps prevent you from becoming infected with HIV.

1. **Before today, have you ever heard about PEP?**

No

Yes

I don’t know

**SKIP Q.D75-D81, IF RESPONSE TO Q.D74 IS “NO”**

1. **Have you ever received information about this medication?**

No

Yes

I don’t know

**SKIP Q.D76, IF RESPONSE TO Q.D75 IS “NO”**

1. **If yes, where did you get information on how to use PEP?**

Pharmacist
 Doctor
 Friends
 HIV counselor

Social media, please specify: _______________
 Other: _______________

**SKIP Q.D77-D81, IF RESPONSE TO Q.C32 IS “HIV” OR “AIDS”**

1. **How willing are you to take PEP after a possible exposure to HIV?**

Not at all willing

Not very willing

Somewhat willing

Very willing

I don’t know

RF

1. **Do you know where to get PEP?**

No

Yes, please specify: ___________

I don’t know

1. **How many hours after a possible exposure to HIV should PEP be taken?**

24 hours

48 hours

72 hours

96 hours

I don’t know

1. **Once you start PEP, how many days should you take it in order for it to be effective?**

7 days

14 days

28 days

35 days

I don’t know

1. **During the past 6 months when you were having anal or frontal sex, did you take post- exposure prophylaxis (PEP) to prevent the transmission of HIV?**

No Yes I have not had anal or frontal sex

**Section E. Social Support**

***This section is INTERVIEWER-ADMINISTERED***

Please tell me how much you disagree or agree with the following statements. **(CARD 20)**

**Perceived social support**

1. **There is a special person who is around when I am in need.**

Very Strongly Disagree
Strongly Disagree
Mildly Disagree
Neutral
Mildly Agree
Strongly Agree
Very Strongly Agree

1. **There is a special person with whom I can share my joys and sorrows**

Very Strongly Disagree
Strongly Disagree
Mildly Disagree
Neutral
Mildly Agree
Strongly Agree
Very Strongly Agree

1. **My family really tries to help me.**

Very Strongly Disagree
Strongly Disagree
Mildly Disagree
Neutral
Mildly Agree
Strongly Agree
Very Strongly Agree

1. **I get the emotional help and support I need from my family.**

Very Strongly Disagree
Strongly Disagree
Mildly Disagree
Neutral
Mildly Agree
Strongly Agree
Very Strongly Agree

1. **I have a special person who is a real source of comfort to me.**

Very Strongly Disagree
Strongly Disagree
Mildly Disagree
Neutral
Mildly Agree
Strongly Agree
Very Strongly Agree

1. **My friends really try to help me.**

Very Strongly Disagree
Strongly Disagree
Mildly Disagree
Neutral
Mildly Agree
Strongly Agree
Very Strongly Agree

1. **I can count on my friends when things go wrong.**

Very Strongly Disagree
Strongly Disagree
Mildly Disagree
Neutral
Mildly Agree
Strongly Agree
Very Strongly Agree

1. **I can talk about my problems with my family.**

Very Strongly Disagree
Strongly Disagree
Mildly Disagree
Neutral
Mildly Agree
Strongly Agree
Very Strongly Agree

1. **I have friends with whom I can share my joys and sorrows.**

Very Strongly Disagree
Strongly Disagree
Mildly Disagree
Neutral
Mildly Agree
Strongly Agree
Very Strongly Agree

1. **There is a special person in my life who cares about my feelings.**

Very Strongly Disagree
Strongly Disagree
Mildly Disagree
Neutral
Mildly Agree
Strongly Agree
Very Strongly Agree

1. **My family is willing to help me make decisions.**

Very Strongly Disagree
Strongly Disagree
Mildly Disagree
Neutral
Mildly Agree
Strongly Agree
Very Strongly Agree

1. **I can talk about my problems with my friends.**

Very Strongly Disagree
Strongly Disagree
Mildly Disagree
Neutral
Mildly Agree
Strongly Agree
Very Strongly Agree

**Transgender, GNC, Non-binary Family Support**

The following questions are about how your family supports your gender identity.

1. **How supportive do you feel your family (parents and/or siblings) is regarding your**

**gender identity?**

Not at all supportive

Unsupportive

Somewhat unsupportive

Neutral

Somewhat supportive

Supportive

Extremely supportive

I have not yet disclosed to my family

1. **How supportive do you feel your immediate family (partner, children, etc.) is regarding your gender identity?**

Not at all supportive

Unsupportive

Somewhat unsupportive

Neutral

Somewhat supportive

Supportive

Extremely supportive

I have not yet disclosed to my family

**Transgender, GNC, Non-binary Peer Support**

The following questions are about your peer group and others living near you.

1. **What portion of your social time is spent with transgender, gender nonconforming or non-binary people?**

None at all

Rarely

Occasionally

Sometimes

Frequently

Most of the time

All of the time

1. **How often have you felt like you were the only transgender, gender nonconforming or non-binary person in the area where you live?**

None at all

Rarely

Occasionally

Sometimes

Frequently

Most of the time

All of the time

**Section F. Gender Identity**

***This section is INTERVIEW-ADMINISTERED***

**Perception of Stigma Associated with Transgender, Gender nonconforming or Non-binary People**

The following questions are about how you perceive other people's reactions to transgender people. We understand that different people have different reactions to a person’s gender identity. When you answer the questions below, think about what happens generally.

1. **Society still shames people for being transgender, gender nonconforming or non-binary.**

Strongly Agree

Agree

Neither Agree nor Disagree

Disagree

Strongly Disagree

1. **Most people have negative reactions to transgender, gender nonconforming or non-binary people.**

Strongly Agree

Agree

Neither Agree nor Disagree

Disagree

Strongly Disagree

1. **Discrimination against transgender, gender non-conforming or non-binary people is still common.**

Strongly Agree

Agree

Neither Agree nor Disagree

Disagree

Strongly Disagree

1. **Only a few people discriminate against transgender, gender nonconforming or non-binary people.**

Strongly Agree

Agree

Neither Agree nor Disagree

Disagree

Strongly Disagree

1. **I worry about aging.**

Strongly Agree

Agree

Neither Agree nor Disagree

Disagree

Strongly Disagree

1. **I worry about becoming unattractive.**

Strongly Disagree

Disagree

Neither Agree nor Disagree

Agree

Strongly Agree

**Gender-Related Fears**

The following questions are about potential fears related to your gender identity.

1. **I fear abandonment if I told others about my gender identity**

Strongly Disagree

Disagree

Neither Agree nor Disagree

Agree

Strongly Agree

1. **I fear loss of my job if my employers knew about my gender identity**

Strongly Disagree

Disagree

Neither Agree nor Disagree

Agree

Strongly Agree

1. **I am afraid to tell my family (parents, grandparents, siblings) about my gender identity**

Strongly Disagree

Disagree

Neither Agree nor Disagree

Agree

Strongly Agree

1. **I fear discrimination**

Strongly Disagree

Disagree

Neither Agree nor Disagree

Agree

Strongly Agree

1. **My family would be accepting and supportive of my gender identity**

Strongly Disagree

Disagree

Neither Agree nor Disagree

Agree

Strongly Agree

I don’t know

**Section G. Substance Use**

***This section is SELF-ADMINISTERED***

This next section will be about substance use and sexual activity. I'll hand over the laptop to you again so you can complete these questions. All of your answers will be kept confidential and will not be used against you in any way or form, so please answer honestly.

Some of the questions will ask you to recall what you've done in your lifetime or what you've done in the past 6 months. To help you answer these questions, 6 months ago was [SELECT DATE]. Did anything, like a birthday or celebration, happen around that time?

Additionally, some questions will ask you to recall things that you've done in the past 30 days. To help you answer these questions, 30 days was [SELECT DATE]. Did anything else, like a birthday or celebration, happen around that time?

## Alcohol

The following questions are about your alcohol use in the past 6 months. Please read each question carefully and select the response that is most relevant to your alcohol use in the past 6 months.

1. **How often do you have a drink containing alcohol?**

Never

Monthly or less

- 1. times a month

2-3 times a week
4 or more times a week

1. **How old were you when you first got drunk or buzzed?**  ______________

I have never drank alcohol

**SKIP Q.G3-G11 IF RESPONSE TO Q.G2 IS “I HAVE NEVER DRANK ALCOHOL”**

1. **How many drinks containing alcohol do you have on a typical day when you are drinking?**

1 or 2

3 or 4

5 or 6

7 to 9

10 or more

1. **How often do you have six or more drinks on one occasion?**

Never

Less than monthly

Monthly

Weekly

Daily or almost daily

1. **How often during the past 6 months have you found that you were not able to stop drinking once you started?**

Never

Less than monthly

Monthly

Weekly

Daily or almost daily

1. **How often during the past 6 months have you failed to do what was normally expected of you because of drinking?**

Never

Less than monthly

Monthly

Weekly

Daily or almost daily

1. **How often during the past 6 months have you needed a first drink in the morning to get yourself going after a heavy drinking session?**

Never

Less than monthly

Monthly

Weekly

Daily or almost daily

1. **How often during the past 6 months have you had a feeling of guilt or remorse after drinking?**

Never

Less than monthly

Monthly

Weekly

Daily or almost daily

1. **How often during the past 6 months have you been unable to remember what happened the night before because of your drinking?**

Never

Less than monthly

Monthly

Weekly

Daily or almost daily

1. **Have you or someone else been injured because of your drinking?**

No

Yes, but not in the last 6 months

Yes, during the last 6 months

1. **Has a relative, friend, doctor or other health care worker been concerned about your drinking or suggested you cut down?**

No

Yes, but not in the last 6 months

Yes, during the last 6 months

## Family History - Alcohol

Now I would like to ask you a question about whether any of your biological relatives, regardless of whether or not they are now living, have EVER been alcoholics or problem drinkers. By alcoholic or problem drinker, I mean a person who has physical or emotional problems because of drinking; problems with a spouse/partner, family, or friends because of drinking; problems at work or school because of drinking; problems with the police because of drinking - like drunk driving or a person who seems to spend a lot of time drinking or being hung over.

1. **Have any of your biological relatives been an alcoholic or problem drinker at any time in his/her life?**

No

Yes

I don’t know

**SKIP Q.G13, IF RESPONSE TO Q.G12 IS “NO”**

1. **Please indicate which biological relatives have been alcoholics or problem drinkers at ANY time in his/her life. Select all that apply.**

Parent

Grandparent

Brother or Sister

Uncle or Aunt

First Cousins

Other (please describe): _________________

I’m not sure

##

## Patterns of Substance Use - Nicotine

The next few questions will be about your experience with smoking cigarettes. Please do not think about e-cigarette use while answering the following questions. There is a separate section on e-cigarettes that will come later.

1. **Have you ever smoked cigarettes?**

Never

Once or Twice

Occasionally but not regularly

Regularly in the past

Regularly now

**SKIP Q.G15-G18, IF ANSWER TO Q.G14 IS “NEVER”**

1. **How old were you when you started smoking cigarettes?** ___________
2. **About how many cigarettes have you smoked in your entire life?**

500 or more

100 or more cigarettes (5 or more packs)

26 to 99 cigarettes (more than one pack, but less than 5 packs)

16 to 25 cigarettes (about 1 pack total)

6 to 15 cigarettes (about ½ pack total)

2 to 5 cigarettes

1 cigarette

1 or more puffs, but never a whole cigarette

1. **When was the last time you smoked a cigarette, even a puff?**

In the last 24 hours

Not in the last 24 hours, but sometime during the past 7 days

Not during the past 7 days, but sometime the PAST 30 DAYS

Not during the past 30 days, but sometime during the PAST 6 MONTHS

Not during the PAST 6 MONTHS, but sometime during the past year

1 to 4 years ago

5 or more years ago

1. **How frequently have you smoked cigarettes during the past 30 days?**

Less than one cigarette per day

One to five cigarettes per day

About one-half pack per day

About one pack per day

About one and one-half packs per day

Two packs or more per day

The next few questions will be about your experience with e-cigarettes. Please do not think about traditional cigarettes while answering the following question.

1. **Have you ever smoked e-cigarettes (electronic cigarettes; brands include NJOY, Blu, Premium, Smoke Tip, Safe Cig, and Duo Pro)?**

Never

Once or Twice

Occasionally but not regularly

Regularly in the past

Regularly now

**SKIP Q.G20-G23, IF ANSWER TO Q.G19 IS “NEVER”**

1. **How old were you when you started smoking e-cigarettes?** __________
2. **How many cartridges or disposable e-cigarettes have you used in your entire life?**

500 or more cartridges

100 or more cartridges

26 to 99 cartridges

16 to 25 cartridges

6 to 15 cartridges

2 to 5 cartridges

1 cartridge

1 or more puffs, but never a whole cartridge

1. **When was the last time you smoked an e-cigarette, even a puff?**

In the last 24 hours

Not in the last 24 hours, but sometime during the past 7 days

Not during the past 7 days, but sometime the PAST 30 DAYS

Not during the past 30 days, but sometime during the PAST 6 MONTHS

Not during the PAST 6 MONTHS, but sometime during the past year

1 to 4 years ago

5 or more years ago

1. **How frequently have you smoked e-cigarettes during the past 30 days?**

Less than one e-cigarette cartridge per day

One to five e-cigarette cartridges per day

About 10 e-cigarette cartridges per day

About 20 e-cigarette cartridges per day

About 30 e-cigarette cartridges per day

40 or more e-cigarette cartridges per day

## Non-Prescription Drugs

The next set of questions is about non-prescription drug use. Your answers will be kept confidential and will not be used against you in any way or form so please answer honestly.

1. **In your lifetime, have you ever used any of the following non-prescription drugs? Please select all that apply**.

Marijuana (also called ‘Weed’ or ‘Pot’)

Synthetic marijuana (also called ‘K2’ or ‘Spice’)

Cocaine or crack (also called ‘Coke’, ‘Snow’, ‘Blow’, ‘Rock’ or ‘Freebase’)

Heroin (also called ‘Smack’, ‘Junk’, ‘Black tar’ or ‘China white’)

Fentanyl (also called ‘Apache’, ‘China Girl’, ‘China White,’ ‘Dance Fever’ or ‘Jackpot’)

Ecstasy (also called ‘Molly,’ ‘E,’ or ‘MDMA’)

Methamphetamines (also called ‘Meth’, ‘Crystal meth’, ‘Crank’ or ‘Speed’)

GHB (also called ‘G’, ‘Liquid G’, ‘Liquid E’ or ‘Liquid Ecstasy’)

Ketamine (also called ‘K’, ‘Special K’ or ‘Vitamin K’)

Poppers (also called ‘Snappers’ or ‘Liquid gold’)

Inhalants (such as glue, nail polish remover, gasoline, solvents, butane, propellants, or whippets)

Hallucinogens or psychedelics (also called ‘PCP’, ‘LSD’, ‘Acid’ or ‘Mushrooms’)

Other drug(s): _____________

I have never used any of these drugs

**SKIP Q.G25-G26, IF ANSWER TO Q.G24 IS “I HAVE NEVER USED ANY OF THESE DRUGS”**

1. **How old were you when you first used any type of non-prescription drugs?**
    Marijuana (also called ‘Weed’ or ‘Pot’) _________

I have never used this drug

Synthetic marijuana (also called ‘K2’ or ‘Spice’) _________

I have never used this drug

Cocaine or crack (also called ‘Coke’, ‘Snow’, ‘Blow’, ‘Rock’ or ‘Freebase’) _________

I have never used this drug

Heroin (also called ‘Smack’, ‘Junk’, ‘Black tar’ or ‘China white’) _________

I have never used this drug

Fentanyl (also called ‘Apache’, ‘China Girl’, ‘China White,’ _________

‘Dance Fever’ or ‘Jackpot’)

I have never used this drug

Ecstasy (also called ‘Molly,’ ‘E,’ or ‘MDMA’) _________

I have never used this drug

Methamphetamines (also called ‘Meth’, ‘Crystal meth’, ‘Crank’ or ‘Speed’) _________

I have never used this drug

GHB (also called ‘G’, ‘Liquid G’, ‘Liquid E’ or ‘Liquid Ecstasy’) _________

I have never used this drug

Ketamine (also called ‘K’, ‘Special K’ or ‘Vitamin K’) _________

I have never used this drug

Poppers (also called ‘Snappers’ or ‘Liquid gold’) _________

I have never used this drug

Inhalants (such as glue, nail polish remover, gasoline, solvents, _________
butane, propellants, or whippets)

I have never used this drug

Hallucinogens or psychedelics (also called ‘PCP’, _________
‘LSD’, ‘Acid’ or ‘Mushrooms’)

I have never used this drug

When answering the following question only think about the **past 6 months**, the time period between today and [date6m], or around ([event6m]).

1. **In the past 6 months, have you used any of the following non-prescription drugs? Please select all that apply.**

Marijuana (also called ‘Weed’ or ‘Pot’)

Synthetic marijuana (also called ‘K2’ or ‘Spice’)

Cocaine or crack (also called ‘Coke’, ‘Snow’, ‘Blow’, ‘Rock’ or ‘Freebase’)

Heroin (also called ‘Smack’, ‘Junk’, ‘Black tar’ or ‘China white’)

Fentanyl (also called ‘Apache’, ‘China Girl’, ‘China White,’ ‘Dance Fever’ or ‘Jackpot’)

Ecstasy (also called ‘Molly,’ ‘E,’ or ‘MDMA’)

Methamphetamines (also called ‘Meth’, ‘Crystal meth’, ‘Crank’ or ‘Speed’)

GHB (also called ‘G’, ‘Liquid G’, ‘Liquid E’ or ‘Liquid Ecstasy’)

Ketamine (also called ‘K’, ‘Special K’ or ‘Vitamin K’)

Poppers (also called ‘Snappers’ or ‘Liquid gold’)

Inhalants (such as glue, nail polish remover, gasoline, solvents, butane, propellants, or whippets)

Hallucinogens or psychedelics (also called ‘PCP’, ‘LSD’, ‘Acid’ or ‘Mushrooms’)

Other drug(s): _____________

I have not used any of these drugs in the **PAST 6 MONTHS**

**Q.G27-G38: PARTICIPANTS WHO SELECTED MARIJUANA IN Q.G26 ONLY (INCLUDE THOSE WHO HAVE USED MEDICAL MARIJUANA FROM Q.C94)**

1. **What are some of the reasons that you've used marijuana in the past 6 months? [Check all that apply]**

To experiment

To have fun

To celebrate

To help relax or feel more confident

To help think differently or creatively

To be in my own world

To relieve boredom

To help me focus

To help sleep

To relieve physical pain

To relieve feeling uptight or anxious

To cope with feeling depressed

To cope with some other problem or problems

Felt pressure from others to do it

Instead of alcohol or as a replacement for alcohol

Substitution/replacement for other drugs besides alcohol

I think marijuana is a low risk drug compared to other drugs

Due to the availability of marijuana

For spiritual reasons

Other: ­­­________________

I don't know

Refuse to answer

The next set of questions is about your use of prescription drugs.

Prescription stimulants or amphetamines are sometimes prescribed by doctors for people who have trouble paying attention, are hyperactive, have ADHD, or have trouble staying awake. They are sometimes called uppers, ups, pep pills, and include drugs like Adderall and Ritalin. These stimulants do not include medications that can be bought in drug stores or grocery stores without a prescription.

1. **In your lifetime, have you ever used any type of prescription stimulant such as Adderall, Concerta, Dexedrine, Ritalin or Vyvanse?**

No

Yes

1. **You said that you have used prescription stimulants in your lifetime. Were these prescribed to you by a healthcare professional?**

No

Yes

1. **In the past 6 months, have you used any type of prescription stimulant such as Adderall, Concerta, Dexedrine, Ritalin or Vyvanse?**

No

Yes

1. **You said that you have used prescription stimulants in the past 6 months. Were these prescribed to you by a healthcare professional?**

No

Yes

Prescription painkillers are sometimes prescribed by health care professionals to relieve people of pain and include drugs such as Vicodin, Codeine, Percocet, Hydrocodone, Morphine or OxyContin. We are not interested in your use of over-the-counter pain relievers such as Aspirin, Tylenol, or Advil that can be bought in drug stores or grocery stores without a prescription.

1. **In your lifetime, have you ever used prescription painkillers such as Vicodin, Codeine, Percocet, Hydrocodone, Morphine, or OxyContin?**

No

Yes

1. **You said that you have used prescription painkillers in your lifetime. Were these prescribed to you by a healthcare professional?**

No

Yes

1. **In the past 6 months, did you use prescription painkillers such as Vicodin, Codeine, Percocet, Hydrocodone, Morphine, or OxyContin?**

No

Yes

1. **You said that you have used prescription painkillers in the past 6 months. Were these prescribed to you by a healthcare professional?**

No

Yes

Prescription depressants or tranquilizers are sometimes prescribed by doctors to calm people down, quiet their nerves, relieve anxiety or relax their muscles. Prescription depressants are sometimes called downers and include medications such as Ativan, Klonopin, Librium, Valium or Xanax. These depressants do not include medications that can be bought in drug stores or grocery stores without a prescription.

1. **In your lifetime, have you ever used any type of prescription depressant or tranquilizer such as Ativan, Klonopin, Librium, Valium or Xanax?**

No

Yes

1. **You said that you have used prescription depressants or tranquilizers in your lifetime. Were these prescribed to you by a healthcare professional?**

No

Yes

1. **In the past 6 months, have you used any type of prescription depressant or tranquilizer such as Ativan, Klonopin, Librium, Valium or Xanax?**

No

Yes

1. **You said that you have used prescription depressants or tranquilizers in the past 6 months. Were these prescribed to you by a healthcare professional?**

No

Yes

Erectile medications are sometimes prescribed by doctors for people who have trouble getting or sustaining an erection and include drugs such as Viagra, Cialis or Levitra.

1. **In your lifetime, have you ever used any type of erectile medication such as Viagra, Cialis or Levitra?**

No

Yes

1. **You said that you have used erectile medications in your lifetime. Were these prescribed to you by a healthcare professional?**

No

Yes

1. **In the past 6 months, have you used any type of erectile medication such as Viagra, Cialis or Levitra?**

No

Yes

1. **You said that you have used erectile medications in the past 6 months. Were these prescribed to you by a healthcare professional?**

No

Yes

You indicated using the following drugs in the **past 6 months**. Please tell us how many days it has been since you last used each of the drugs listed below. If you are having a difficult time remembering, please provide us with your best estimate.

**SKIP Q.G44-G82, IF RESPONSE TO Q.G26 IS “I HAVE NOT USED ANY OF THESE DRUGS IN THE PAST 6 MONTHS”**

1. **Marijuana (also called ‘Weed’ or ‘Pot’): __________ (Days)**
2. **Cocaine or crack (also called ‘Coke’, ‘Snow’, ‘Blow’, ‘Rock’ or ‘Freebase’):
    __________ (Days)**
3. **Heroin (also called ‘Smack’, ‘Junk’, ‘Black tar’ or ‘China white’): __________ (Days)**
4. **Fentanyl (also called ‘Apache’, ‘China Girl’, ‘China White,’ ‘Dance Fever’ or ‘Jackpot’): __________ (Days)**
5. **Ecstasy (also called ‘Molly,’ ‘E,’ or ‘MDMA’): __________ (Days)**
6. **Methamphetamines (also called ‘Meth’, ‘Crystal meth’, ‘Crank’ or ‘Speed’):
    __________ (Days)**
7. **Prescription stimulants or amphetamines such as Adderall, Concerta, Dexedrine, Ritalin or Vyvanse: __________ (Days)**
8. **Prescription painkillers such as Vicodin, Codeine, Percocet, Hydrocodone, Morphine or OxyContin: __________ (Days)**
9. **Prescription depressants or tranquilizers such as Ativan, Klonopin, Librium, Valium or Xanax: __________ (Days)**

**Patterns of Substance Use - Illicit Drugs**

You indicated using the following drugs in the past 6 months. Please tell us on how many occasions you have used each drug in the past 30 days, the time period between today and [date30d], or around ([event30d]). If you are having a difficult time remembering, please provide us with your best estimate. Remember, your answers will be kept confidential.

****Pipe in responses from Q.G44-Q.G52****

1. **On how many occasions have you used cocaine or crack (also called ‘Coke’, ‘Snow’, ‘Blow’, ‘Rock’ or ‘Freebase’) in the past 30 days?**

0

1 - 2

3 - 5

6 - 9

10 - 19

20 - 39

40 or More

1. **On how many occasions have you used heroin (also called ‘Smack’, ‘Junk’, ‘Black tar’ or ‘China white’) in the past 30 days?**

0

1 - 2

3 - 5

6 - 9

10 - 19

20 - 39

40 or More

1. **On how many occasions have you used Ecstasy (also called ‘Molly,’ ‘E,’ or ‘MDMA’) in the past 30 days?**

0

1 - 2

3 - 5

6 - 9

10 - 19

20 - 39

40 or More

1. **On how many occasions have you used Methamphetamines (also called ‘Meth’, ‘Crystal meth’, ‘Crank’ or ‘Speed’) in the past 30 days?**

0

1 - 2

3 - 5

6 - 9

10 - 19

20 - 39

40 or More

1. **On how many occasions have you used prescription stimulants or amphetamines such as Adderall, Concerta, Dexedrine, Ritalin or Vyvanse in the past 30 days?**

0

1 - 2

3 - 5

6 - 9

10 - 19

20 - 39

40 or More

1. **On how many occasions have you used prescription painkillers such as Vicodin, Codeine, Percocet, Hydrocodone, Morphine or OxyContin in the past 30 days?**

0

1 - 2

3 - 5

6 - 9

10 - 19

20 - 39

40 or More

1. **On how many occasions have you used prescription depressants or tranquilizers such as Ativan, Klonopin, Librium, Valium or Xanax in the past 30 days?**

0

1 - 2

3 - 5

6 - 9

10 - 19

20 - 39

40 or More

1. **On how many occasions have you used fentanyl (also called ‘Apache’, ‘China Girl’, ‘China White,’ ‘Dance Fever’ or ‘Jackpot’) in the past 30 days?**

0

1 - 2

3 - 5

6 - 9

10 - 19

20 - 39

40 or More

1. **What are the different ways that you used fentanyl in the last 30 days? [Choose all that apply]**

Lollipop

Pill (oral tablet)

Oral spray

Patch

Injection

Mixed with other drugs

Accidentally (you didn’t know it was mixed with another drug)

Other, please specify: ____________________

Don't know

Refuse to answer

**ASK Q.G62 ONLY IF ANSWER TO Q.G61 IS “MIXED WITH OTHER DRUGS”**

1. **Which drug was the fentanyl mixed with? (Select all that apply)**

Marijuana (also called ‘Weed’ or ‘Pot’)

Synthetic marijuana (also called ‘K2’ or ‘Spice’)

Cocaine or crack (also called ‘Coke’, ‘Snow’, ‘Blow’, ‘Rock’ or ‘Freebase’)

Heroin (also called ‘Smack’, ‘Junk’, ‘Black tar’ or ‘China white’)

Ecstasy (also called ‘Molly,’ ‘E,’ or ‘MDMA’)

Methamphetamines (also called ‘Meth’, ‘Crystal meth’, ‘Crank’ or ‘Speed’)

GHB (also called ‘G’, ‘Liquid G’, ‘Liquid E’ or ‘Liquid Ecstasy’)

Ketamine (also called ‘K’, ‘Special K’ or ‘Vitamin K’)

Poppers (also called ‘Snappers’ or ‘Liquid gold’)

Inhalants (such as glue, nail polish remover, gasoline, solvents, butane, propellants, or whippets)

Hallucinogens or psychedelics (also called ‘PCP’, ‘LSD’, ‘Acid’ or ‘Mushrooms’)

Other drug(s): _____________

I don’t know

1. **On how many occasions have you used marijuana (also called ‘Weed’ or ‘Pot’) in the past 30 days?**

0

1 - 2

3 - 5

6 - 9

10 - 19

20 - 39

40 or More

**SKIP Q.G64-G92, IF ANSWER TO Q.G63 IS “0”**

1. **What are the different ways that you used marijuana in the last 30 days? [Choose all that apply]**

Pipe/Bowl

Bong

Vaporizer for bud/flower (volcano, pax)

Electronic vaporizer for oils (e-joint, vape pen)

Oil rig for concentrates (dabs)

Joint

Blunt

Skiff or spliff (marijuana and tobacco mixed together)

Edible form

Beverage form

Other, please specify: ____________________

Don't know

Refuse to answer

1. **What was your most preferred way to use marijuana in the last 30 days?**

Pipe/Bowl

Bong

Vaporizer for bud/flower (volcano, pax

Electronic vaporizer for oils (e-joint, vape pen)

Oil rig for concentrates (dabs)

Joint

Blunt

Skiff or spliff (marijuana and tobacco mixed together)

Edible form

Beverage form

Other, please specify: ____________________

Don't know

I don’t have a preference

Refuse to answer

1. **Have you sold marijuana in the past 30 days?**

Yes – sold to make a profit

Yes – sold largely at cost (you sold your marijuana for the same amount that you bought it)

No

Refuse to answer

1. **How much money did you typically spend on marijuana products in the last 30 days?**

______________ (numeric value) [dollars] (Don't Know=777, Refuse To Answer=888)

**Medical and Recreational Marijuana Use**

**Recreational marijuana:** Using marijuana to socialize with others, to increase creativity, or to make experiences more pleasurable, interesting, or exciting.

**Medical/medicinal marijuana:** Using marijuana to treat or help cope with any physical issues, such as pain or discomfort, or psychological conditions, such as feeling anxious or sad, insomnia, etc.

1. **How often did you use marijuana for medical purposes in the last 30 days?**

Not at all or very minor medicinal use

Medical use for a small portion of the time

Medical use half the time

Medical use more than half of the time

Medical use almost all of the time

1. **How often did you use marijuana for recreational purposes in the last 30 days?**

Not at all or very minor recreational use

Recreational use for a small portion of the time

Recreational use half the time

Recreational use more than half of the time

Recreational use almost all of the time

**Marijuana Use Problems**

You indicated that you have used marijuana in **the past 6 months**. Have you experienced any of the following related to your marijuana use during **the past 6 months**?

**SKIP Q.G70-G82, IF RESPONSE TO Q.G26 IS *NOT* “SYNTHETIC MARIJUANA” OR “MARIJUANA”**

1. **Have job or school troubles as a result of your marijuana use—like missing too much work, not doing your work well, being demoted or losing a job, or being suspended, expelled or dropping out of school?**

No Yes

1. **Continue to use marijuana even though you knew it was causing you trouble with your family or friends? (Social/interpersonal problems related to use)**

No Yes

1. **More than once drive a car, motorcycle, truck, boat, or other vehicle when you were under the influence of marijuana? - Hazardous use**

No Yes

1. **Find yourself under the influence of marijuana or feeling its aftereffects in situations that increased your chances of getting hurt—like swimming, using machinery, or walking in a dangerous area or around heavy traffic? – Hazardous use**

No Yes

1. **Get arrested, get held at a police station or have any other legal problems because of your marijuana use?**

No Yes

1. **Find that you had to use much more marijuana than you once did to get the effect you wanted?**

No Yes

1. **Have any of the following bad aftereffects when the effects of marijuana were wearing off, such as sweating, muscle aches, shaking, or bad headaches? This includes the morning after using it or in the first few days after stopping or cutting down on it?**

No Yes

1. **Often use marijuana in larger amounts or for a much longer period than you meant to?**

No Yes

1. **More than once try to stop or cut down on using marijuana but found you couldn’t do it?**

No Yes

1. **Give up or cut down on activities that were important to you in order to use marijuana— like work, school, or associating with friends or relatives?**

No Yes

1. **Have a period when you spent a lot of time making sure you always had enough marijuana available?**

No Yes

1. **Continue to use marijuana even though you knew it was causing you a health problem or making a health problem worse?**

No Yes

1. **Have you had a craving or a strong desire or urge to use marijuana?**

No Yes

**Injection Drug Use**

**In the following section I will be asking you questions about your experiences with injecting various substances.**

1. **Have you ever injected any drugs like heroin, cocaine, crystal, ketamine, etc.?**

No Yes I don’t know

**SKIP Q.G84-G93, IF RESPONSE TO Q.G83 IS “NO”**

1. **Which drugs you inject:_________________________**
2. **At what age did you first inject any drug?_____________ years old**
3. **Have you ever injected any other substances like hormones or steroids?**

No Yes I don’t know

**SKIP Q.G87-G88, IF RESPONSE TO Q.G86 IS “NO”**

1. **Which substances like hormones or steroids you inject:________________**
2. **At what age did you first inject any substance?_____________ years old**
3. **Where did you get your needles? (Check all that apply)**

Needle exchange

Pharmacy

A friend

Shooting gallery

Found a used needle

Reused my own needle

Bought it on the street

Other: **________________**

1. **Have you ever used the same needle as someone else?**

No Yes I don’t know

**SKIP Q.G91-G92, IF RESPONSE TO Q.G90 IS “NO”**

1. **In thinking about the time(s) that you injected over the past three months, how often did**

**you use the same needles/syringe that someone else had already used? Would you say…**

Never

Less than half the time

Half the time

More than half the time

Every time

**SKIP Q.G92, IF RESPONSE TO Q.G91 IS “NEVER”**

1. **Of those times, how often was the needle/syringe cleaned with bleach before you used it?**

Never

Less than half the time

Half the time

More than half the time

Every time

1. **Imagine you are in a situation where in order to inject you would have to use the same**

**needle that someone else has used. How confident are you that you could clean the needle/syringe with bleach before you used it? Would you say...**

Not confident at all

Not very confident

Somewhat confident

Very confident

**Section H. Discrimination**

***This section is INERVIEWER-ADMINISTERED***

**Racism and Life Experience**

The following questions are about your experience of racism in a variety of situations over the past year.

| **In the past 12 months…** | **Never** | **Rarely** | **Sometimes** | **Fairly Often** | **All of the Time** | **I Don’t Know** |
| --- | --- | --- | --- | --- | --- | --- |
| 1. How often have your civil rights been violated (i.e., job or housing discrimination due to   racism, racial discrimination, or racial prejudice)? |  |  |  |  |  |  |
| 1. How often have others said or acted as if you are oversensitive or paranoid about racism? |  |  |  |  |  |  |
| 1. How often have you witnessed prejudice or discrimination directed at someone else because of their race/ethnic group? |  |  |  |  |  |  |
| 1. How often have others reacted to you as if they were afraid or intimidated of you because of your race? |  |  |  |  |  |  |
| 1. How often have you been observed or followed while in public places because of your race/ethnic group? |  |  |  |  |  |  |
| 1. How often have you been treated as if you were “stupid” or “talked down to” because of your race/ethnic group? |  |  |  |  |  |  |
| 1. How often have your ideas or opinions been minimized, ignored, or devalued because of your race/ethnic group? |  |  |  |  |  |  |
| 1. How often have you heard (or been told) a racially offensive or insensitive comment or joke? |  |  |  |  |  |  |
| 1. How often have you been mistaken for someone who serves others (i.e., janitor, maid) because of your race/ethnic group? |  |  |  |  |  |  |
| 1. How often have you been mistaken for someone else of your same race/ethnicity (who may not look like you at all)? |  |  |  |  |  |  |

**Discrimination and Harassment**

The following questions are about any discrimination or harassment you've experienced related to

being transgender, gender nonconforming, or non-binary -- whether from police, in school, or in a prison environment.

**Have you done any of the following to avoid discrimination because of your gender identity? If you are/were not employed, mark not applicable.**

|  | **Not applicable** | **No** | **Yes** |
| --- | --- | --- | --- |
| 1. **Stayed in a job I'd prefer to leave** |  |  |  |
| 1. **Didn't seek a promotion or a raise** |  |  |  |
| 1. **Changed jobs** |  |  |  |
| 1. **Delayed my gender transition** |  |  |  |
| 1. **Hid my gender identity or gender transition** |  |  |  |
| 1. **I have not done anything to avoid discrimination** |  |  |  |

**Because of your gender identity, which of the following experience have you had at work?**

|  | **Not applicable** | **No** | **Yes** |
| --- | --- | --- | --- |
| 1. **I feel more comfortable and my performance has improved** |  |  |  |
| 1. **I did not get a job I applied for because of being transgender or gender nonconforming** |  |  |  |
| 1. **I am or have been underemployed, that is working in the field I should not be in or a position for which I am overqualified** |  |  |  |
| 1. **I was removed from direct contact with clients, customers or patients** |  |  |  |
| 1. **I was denied a promotion** |  |  |  |
| 1. **I lost my job** |  |  |  |
| 1. **I was harassed by someone at work** |  |  |  |
| 1. **I was the victim of physical violence by someone at work** |  |  |  |
| 1. **I was the victim of sexual assault by someone at work** |  |  |  |
| 1. **I was forced to present in the wrong gender to keep my job** |  |  |  |
| 1. **I was not able to work out a suitable bathroom situation with my employer** |  |  |  |
| 1. **I was denied access to appropriate bathrooms** |  |  |  |
| 1. **I was asked inappropriate questions about my transgender or surgical status** |  |  |  |
| 1. **I was referred to by the wrong pronoun, repeatedly and on purpose** |  |  |  |
| 1. **Supervisors or coworkers shared information about me that they should not have** |  |  |  |

***These next few questions are SELF-ADMINISTERED***

1. **Because of your gender identity, which of the following experience have you had in your interactions with the police?**

Officers generally have treated me with respect

Officers generally have treated me with disrespect

Officers have harassed me

Officers have physically assaulted me

Officers have sexually assaulted me

1. **As a transgender, gender nonconforming, or non-binary person, how comfortable do you feel seeking help from the police?**

Very comfortable

Somewhat comfortable

Neutral

Somewhat uncomfortable

Very uncomfortable

1. **Because of your gender identity, have you ever been arrested or held in a cell?**

No Yes

1. **Have you ever been sent to jail or prison for any reason?**

No Yes

1. **How long have you been in jail or prison in total?**

Under six months

Six months to a year

One to three years

Three to five years

Five to ten years

Ten or more years

| **If you were jailed or in prison, have you ever experienced any of the following because of your gender identity?** | **Harassed** | **Physically assaulted or attacked** | **Sexually assaulted or attacked** | **Denied Hormones** | **Denied regular medical care** |
| --- | --- | --- | --- | --- | --- |
| 1. From other prisoners |  |  |  |  |  |
| 1. From correctional officers or other prison staff |  |  |  |  |  |

1. **Have you attended school at any level (elementary school or higher) as your current gender identity?**

No Yes

1. **Because of your gender identity, were you a target of harassment, discrimination or violence at elementary school? (select all that apply)**

Did not attend such a school

Not out as transgender or gender-nonconforming at this point

Harassed or bullied by another student(s) (including harassment online)

Harassed or bullied by a teacher, coach, or staff member (including harassment online)

Sexually assaulted or attacked by another student(s)

Sexually assaulted or attacked by teacher or staff member

Physically assaulted or attacked by another student(s)

Physically assaulted or attacked by teachers or staff

Expelled, thrown out, or denied enrollment

Other, please specify: __________________

None of the above

Not applicable, I did not experience negative outcomes

1. **Because of your gender identity, have you been a target of harassment, discrimination or violence at junior high/middle school?**

Did not attend such a school

Not out as transgender or gender nonconforming at this point

Harassed or bullied by students

Harassed or bullied by teachers or staff

Physically assaulted or attacked by students

Physically assaulted or attacked by teachers or staff

Sexually assaulted or attacked by students

Sexually assaulted or attacked by teachers or staff

Expelled, thrown out, or denied enrollment

Not applicable, I did not experience negative outcomes

1. **Because of your gender identity, have you been a target of harassment, discrimination or violence at high school?**

Did not attend such a school

Not out as transgender or gender nonconforming at this point

Harassed or bullied by students

Harassed or bullied by teachers or staff

Physically assaulted or attacked by students

Physically assaulted or attacked by teachers or staff

Sexually assaulted or attacked by students

Sexually assaulted or attacked by teachers or staff

Expelled, thrown out, or denied enrollment

Not applicable, I did not experience negative outcomes

1. **Because of your gender identity, have you been a target of harassment, discrimination or violence at college?**

Did not attend such a school

Not out as transgender or gender nonconforming at this point

Harassed or bullied by students

Harassed or bullied by teachers or staff

Physically assaulted or attacked by students

Physically assaulted or attacked by teachers or staff

Sexually assaulted or attacked by students

Sexually assaulted or attacked by teachers or staff

Expelled, thrown out, or denied enrollment

Not applicable, I did not experience negative outcomes

1. **Because of your gender identity, have you been a target of harassment, discrimination or violence at graduate or professional school?**

Did not attend such a school

Not out as transgender or gender-nonconforming at this point

Harassed or bullied by students

Harassed or bullied by teachers or staff

Physically assaulted or attacked by students

Physically assaulted or attacked by teachers or staff

Sexually assaulted or attacked by students

Sexually assaulted or attacked by teachers or staff

Expelled, thrown out, or denied enrollment

Not applicable, I did not experience negative outcomes

1. **Because of your gender identity, have you been a target of harassment, discrimination or violence at technical school?**

Did not attend such a school

Not out as transgender or gender-nonconforming at this point

Harassed or bullied by students

Harassed or bullied by teachers or staff

Physically assaulted or attacked by students

Physically assaulted or attacked by teachers or staff

Sexually assaulted or attacked by students

Sexually assaulted or attacked by teachers or staff

Expelled, thrown out, or denied enrollment

Not applicable, I did not experience negative outcomes

***These next few questions are INTERVIEWER-ADMINISTERED***

| **Because of your gender identity, which of the following statements are true?** | **Not applicable** | **No** | **Yes** |
| --- | --- | --- | --- |
| 1. I had to leave school because the harassment was so bad |  |  |  |
| 1. I had to leave school for financial reasons related to my gender transition |  |  |  |
| 1. I lost or could not get financial aid or scholarships |  |  |  |
| 1. I was denied housing on campus because of my gender identity |  |  |  |
| 1. I was not provided appropriate housing on campus because of my gender identity |  |  |  |
| 1. I was not allowed to use the appropriate bathrooms at school |  |  |  |

**Gender Discrimination**

The following questions are about any discrimination you've experienced because of your gender identity.

1. **Have you ever been fired from a job because of your gender identity or presentation?**

No Yes

1. **Have you ever experienced problems getting a job because of your gender identity or presentation?**

No Yes

1. **Have you ever been denied or evicted from housing because of your gender identity or presentation?**

No Yes

1. **Have you ever experienced problems getting health or medical services because of your gender identity or presentation?**

No Yes

**Section I. Sexual Activity**

***This section is SELF-ADMINISTERED***

The next set of questions is about your sexual activity with men, women, transgender, gender non-conforming and non-binary partners. This set of questions **does not** refer to occasions where force was used and the activity was against someone’s will. All of your answers are confidential. At this point, feel free to take a 5-minute break if you need to. Just let your interviewer know that you need a break.

We understand that people do not use the same words or names to talk about body parts. Before we start talking about your sexual activity, we want to make sure that we are using words that you are comfortable with.

**Respondent's Gender**

1. **What sex was picked for you at birth?**

Female

Male

1. **What is the most common word you use to describe a flesh penis (not a dildo)?** ________________________
2. **What is the most common word you use to describe a vagina?** ________________________

**Most recent sexual encounter**

Please think about the last person you had sex with (oral, anal, or frontal sex).

1. **How would you describe the gender of this person?**

Man who is not transgender (cisgender man)

Transgender man or person on the transmasculine spectrum

Woman who is not transgender (cisgender women)

Transgender woman or person on the transfeminine spectrum

Non-binary, gender queer, gender non-conforming

Other, please specify: _______________

I DON’T KNOW
RF

**Partner Type**

1. **What was your relationship with this person when you had this sexual encounter? (You can choose only one)**

My primary partner

A consistent casual partner

A single encounter

I DON’T KNOW

RF

- *A* ***primary partner is*** *someone you consider yourself to be in a relationship with (e.g., boyfriend, girlfriend, life partner, etc.).*
- *A* ***consistent casual partner*** *is someone you have sex with on a regular basis but do not consider yourself to be in a relationship with (e.g., fuck buddy, friend with benefits, ex-boyfriend).*
- *A* ***single encounter*** *is someone you may have met briefly and had sex with but do not intend to have sex with him or her again (e.g., one-night stand, hookup, etc.).*

1. **What would you call your relationship to this person?**

Husband/wife/spouse

Boyfriend/girlfriend/partner/significant other

Friend or ex-boyfriend/ex-girlfriend

Hook-up or one-night stand

Casual sex partner/fuck buddy/friends with benefits

Trade

Paying partner (trick, john, date)

Paid partner (sex worker)

Live-in partner

Roommate

Person I’m dating

Other (specify): ________________________

Don’t know

RF

1. **Did you use a condom during this most recent anal or frontal sexual encounter?**

No

Yes

I have not had anal or frontal sex

I don’t know

**SKIP Q.I8, IF RESPONSE TO Q.I7 IS “I HAVE NOT HAD ANAL OR FRONTAL SEX”**

1. **When you have sex with this person, how often do you make sure to use a condom the whole time (you or your partner don’t start having sex before you put on the condom or take it off during sex)?**

Never

Less than half of the time

Half of the time

More than half of the time

Every time (Even if your last sexual encounter was a hook up)

I DON’T KNOW

RF

1. **Where did you meet this person?**

Bar

Dance club

Bathhouse

Sex club

Friend’s house/my house

Social service agency

The beach

A park / public cruising area

Bookstore

A party

Community event

On the street

At work

At school

Social media site (Facebook, Instagram, etc.)

Dating app (Grindr, Jack’d, Tinder, etc.)

Craigslist

Personal ad

Support group meeting

Other (specify): ________________________

I don’t know

RF

1. **Is this person younger, your age or older than you?**

Younger than me

My age

Older than me

I don’t know

RF

1. **What is this person’s ethnicity?**

American Indian / Alaska Native

Asian

Black / African American

Native Hawaiian or other Pacific Islander

Hispanic or Latino

White

Mixed

Other: __________________

I don’t know

RF

1. **Are you having sex with other people other than the person you last had sex with?**
    No
    Yes

I don’t know

RF

1. **How financially well-off is this person in comparison to you? (The person you last had sex with)**

Worse
Slightly worse
About the same
Slightly better

Better

I DON’T KNOW

RF

1. **How long had you known that person before having sex with him/her?**

Less than 1 day

2-7 days

8-30 days

31 days to 2 months

More than 2 months, less than 6 months

More than 6 months, less than 1 year

More than 1 year, less than 2 years

2 years or longer

I DON’T KNOW

RF

1. **How long have you been having sex with this person?**

Less than 1 day

2-7 days

8-30 days

31 days to 2 months

More than 2 months, less than 6 months

More than 6 months, less than 1 year

More than 1 year, less than 2 years

2 years or longer

I DON’T KNOW

RF

1. **Did you binge drink* alcohol two hours before or during your most recent sexual
    encounter?**
    **Binge drinking = 5 or more alcoholic drinks within 2 hours*

No

Yes

Don’t know

1. **Did you take any stimulant drugs (i.e., cocaine, meth, molly) or get high two hours before or during this encounter?**

No

Yes

Don’t know

1. **Did the person you last had sex with binge drink* alcohol two hours before or during sex?**

**Binge drinking = 5 or more alcoholic drinks within 2 hours*

No

Yes

Don’t know

1. **Did the person you last had sex with take any stimulant drugs (i.e., cocaine, meth, molly) or get high two hours before or during sex?**

Yes

No

Don’t know

1. **Are you still having sex with this person?**

No

Yes

I DON’T KNOW

RF

**HIV- only**

1. **How much do you think you can trust this person to protect you from sexually transmitted infections or HIV?**

Don’t trust at all

Sometimes can trust

Completely trust

I DON’T KNOW

RF

**HIV+ only**

1. **How much do you think you can trust this person to protect you from sexually transmitted infections (not including HIV)?**

Don’t trust at all

Sometimes can trust

Completely trust

I DON’T KNOW

RF

**All participants**

1. **Have you discussed your current HIV status with this person?**

No

Yes

I DON’T KNOW

RF

1. **What is the current HIV status of this person?**

HIV negative

HIV positive

HIV status unknown

RF

**SKIP Q.I25, IF ANSWER TO Q.I24 IS “HIV STATUS UNKNOWN”**

1. **How do you know this person’s HIV status?**

They told you

They showed you their test results

You were both HIV tested together

You assume their status is negative

Other (specify): ________________________

I DON’T KNOW

RF

**SKIP Q.I26, IF RESPONSE TO Q.I1 IS “FEMALE”**

1. **Have you ever had a sexual partner that got pregnant after you had sex?** No, never
    Yes, number of times: _____
    I don’t know
    RF

**SKIP Q.I27, IF RESPONSE TO Q.I1 IS “MALE”**

1. **Have you ever been pregnant?**
    No, never
    Yes, number of times: _____
    I don’t know
    RF
2. **In the past 30 days, how many sexual partners have you had?** ____________

**HIV+ only**

1. **How many of these partners were HIV negative? If you are having trouble remembering, please make your best estimate.**

0

1
 2
 3

4
 5-10

11-15

16-25

26-50

51-100

More than 100

I don’t know

**­­­­­­­**

**All participants**

**In the last 30 days...**

Answer scale: *All the time, Most of the time, Some of the time, None of the time, Don't know, Refuse to answer*

**Alcohol and drug use during sex**

1. **Generally, in the last 30 days, did you binge drink alcohol (5 or more alcoholic drinks within 2 hours) two hours before or during sex?**

None of the time

Some of the time

Most of the time

All the time

I don’t know

1. **Generally, in the last 30 days, did you take any drugs or get high two hours before or during sex?**

None of the time

Some of the time

Most of the time

All the time

I don’t know

**SKIP Q.I32, IF ANSWER TO Q.I31 IS “NONE OF THE TIME”**

1. **In general, which drugs did you use two hours before or during sex in the last 30 days? Choose all that apply.**

Marijuana (also called ‘Weed’ or ‘Pot’)

Synthetic marijuana (also called ‘K2’ or ‘Spice’)

Cocaine or crack (also called ‘Coke’, ‘Snow’, ‘Blow’, ‘Rock’ or ‘Freebase’)

Heroin (also called ‘Smack’, ‘Junk’, ‘Black tar’ or ‘China white’)

Fentanyl (also called ‘Apache’, ‘China Girl’, ‘China White,’ ‘Dance Fever’ or ‘Jackpot’)

Ecstasy (also called ‘Molly,’ ‘E,’ or ‘MDMA’)

Methamphetamines (also called ‘Meth’, ‘Crystal meth’, ‘Crank’ or ‘Speed’)

GHB (also called ‘G’, ‘Liquid G’, ‘Liquid E’ or ‘Liquid Ecstasy’)

Ketamine (also called ‘K’, ‘Special K’ or ‘Vitamin K’)

Poppers (also called ‘Snappers’ or ‘Liquid gold’)

Inhalants (such as glue, nail polish remover, gasoline, solvents, butane, propellants - whippets)

Hallucinogens or psychedelics (also called ‘PCP’, ‘LSD’, ‘Acid’ or ‘Mushrooms’)

Prescription stimulants (also called uppers, ups, pep pills, and include drugs like Adderall and Ritalin)

Prescription painkillers (Vicodin, Codeine, Percocet, Hydrocodone, Morphine or OxyContin)

Prescription depressants (Ativan, Klonopin, Librium, Valium or Xanax)-

Erectile medications (Viagra, Cialis, or Levitra)

Other drug(s): _____________

**In the last 30 days...**

*Answer scale: All your partners, Most of your partners, Some of your partners, None of your partners, Don't know, Refuse to answer*

**Partner alcohol and drug use during sex**

1. **Generally, in the last 30 days, did your partner(s) binge drink (5 or more alcoholic drinks within 2 hours) two hours before or during sex?** **____________** None of your partners
    Some of your partners
    Most of your partners
    All your partners
    I don’t know
2. **Generally, in the last 30 days, did your partner(s) take any drugs or get high two hours before or during sex?** ____________
    None of your partners
    Some of your partners
    Most of your partners
    All your partners
    I don’t know

**SKIP Q.I35, IF ANSWER TO Q.I34 IS “NONE OF YOUR PARTNERS”**

1. **In general, which drugs did your partner(s) use two hours before or during sex in the last 30 days? Choose all that apply.**

Marijuana (also called ‘Weed’ or ‘Pot’)

Synthetic marijuana (also called ‘K2’ or ‘Spice’)

Cocaine or crack (also called ‘Coke’, ‘Snow’, ‘Blow’, ‘Rock’ or ‘Freebase’)

Heroin (also called ‘Smack’, ‘Junk’, ‘Black tar’ or ‘China white’)

Fentanyl (also called ‘Apache’, ‘China Girl’, ‘China White,’ ‘Dance Fever’ or ‘Jackpot’)

Ecstasy (also called ‘Molly,’ ‘E,’ or ‘MDMA’)

Methamphetamines (also called ‘Meth’, ‘Crystal meth’, ‘Crank’ or ‘Speed’)

GHB (also called ‘G’, ‘Liquid G’, ‘Liquid E’ or ‘Liquid Ecstasy’)

Ketamine (also called ‘K’, ‘Special K’ or ‘Vitamin K’)

Poppers (also called ‘Snappers’ or ‘Liquid gold’)

Inhalants (such as glue, nail polish remover, gasoline, solvents, butane, propellants - whippets)

Hallucinogens or psychedelics (also called ‘PCP’, ‘LSD’, ‘Acid’ or ‘Mushrooms’)

Prescription stimulants (also called uppers, ups, pep pills, and include drugs like Adderall and Ritalin)

Prescription painkillers (such as Vicodin, Codeine, Percocet, Hydrocodone, Morphine or OxyContin)

Prescription depressants (such as Ativan, Klonopin, Librium, Valium or Xanax)

Erectile medications (Viagra, Cialis, or Levitra)

Other drug(s): _____________

**Condom use in the last 30 days**

For the following questions, please use the scales below where 0% is “Never used a condom” and 100% is “Used a condom all of the time.”

**FOR Q.I36-I38, PIPE IN RESPONSES TO Q.I2-I3**

**In the last 30 days, how often did you use a condom…**

1. **during FRONTAL sex? (your penis in their vagina)** ____________

I have not had frontal sex in the last 30 days OR this does not apply to me

1. **during ANAL insertive sex? (you were the ‘top’)** ____________

I have not had anal insertive sex in the last 30 days OR this does not apply to me

1. **during ANAL receptive sex? (you were the ‘bottom’)** ____________

I have not had anal receptive sex in the last 30 days OR this does not apply to me

**Sex in the last 6 months**

Now, we will ask you questions about people you have slept with in the past 6 months. Consider ALL the people you have had any kind of sex with in the **LAST 6 MONTHS**.

**Total number of partners in last 6 months**

1. **In the last six months, how many sexual partners have you had?** ____________

**SKIP Q.I40-I74, IF ANSWER TO Q.I39 IS “0”**

1. **How many of your sexual partners in the last 6 months were:**

*Enter 0 if you have not had this type of partner in the last 6 months.

Cis men __________

Cis women __________

Trans women __________
 Trans men __________

Other __________
 (please specify, other: --)

**In the last 6 months, with how many:**

*Answer scale: 1, 2, 3, 4, 5-10, 11-15, 16-25, 26-50, 51-100, More than 100, Don't know, Refuse to answer*

**FOR Q.I41-I54, PIPE IN RESPONSES TO Q.I2-I3**

**Filter the following questions based on responses from Q.I40**

1. **CIS MEN have you had ANAL insertive sex with? (you were the ‘top’)** _________

This does not apply to me

1. **CIS MEN have you had ANAL receptive sex with? (you were the ‘bottom’)** _________

1. **CIS WOMEN have you had FRONTAL sex with? (your penis in their vagina)** _________

This does not apply to me

1. **CIS WOMEN have you had ANAL insertive sex with? (your penis in their butt)** _________

This does not apply to me

1. **TRANS WOMEN have you had FRONTAL sex with? (your penis in their vagina)** _________

This does not apply to me

1. **TRANS WOMEN have you had ANAL insertive sex with? (your penis in their butt)**

_________

This does not apply to me

1. **TRANS WOMEN have you had ANAL receptive sex with? (their penis in your butt)** _________
2. **TRANS MEN have you had FRONTAL sex with? (your penis in their vagina)** _________

This does not apply to me

1. **TRANS MEN have you had ANAL insertive sex with? (your penis in their butt)**

_________

This does not apply to me

1. **TRANS MEN have you had ANAL receptive sex with? (their penis in your butt)**
2. **OTHER (insert from above) have you had ANAL insertive sex with? (you were the ‘top’)** _________

This does not apply to me

1. **OTHER have you had ANAL receptive sex with? (you were the ‘bottom’)** _________
2. **OTHER have you had FRONTAL sex with? (your penis in their vagina)** _________

This does not apply to me

**In the last six months...**

**Alcohol and drug use during sex - past 6 months**

1. **Generally, in the last 6 months, did you binge drink alcohol (5 or more alcoholic drinks within 2 hours) two hours before or during sex?**

None of the time

Some of the time

Most of the time

All the time

I don’t know

1. **Generally, in the last 6 months, did you take any drugs or get high two hours before or during sex?**

None of the time

Some of the time

Most of the time

All the time

I don’t know

**SKIP Q.I57, IF ANSWER TO Q.I56 IS “NONE OF THE TIME”**

1. **In general, which drugs did you use two hours before or during sex in the last 30 days? Choose all that apply.**

Marijuana (also called ‘Weed’ or ‘Pot’)

Synthetic marijuana (also called ‘K2’ or ‘Spice’)

Cocaine or crack (also called ‘Coke’, ‘Snow’, ‘Blow’, ‘Rock’ or ‘Freebase’)

Heroin (also called ‘Smack’, ‘Junk’, ‘Black tar’ or ‘China white’)

Fentanyl (also called ‘Apache’, ‘China Girl’, ‘China White,’ ‘Dance Fever’ or ‘Jackpot’)

Ecstasy (also called ‘Molly,’ ‘E,’ or ‘MDMA’)

Methamphetamines (also called ‘Meth’, ‘Crystal meth’, ‘Crank’ or ‘Speed’)

GHB (also called ‘G’, ‘Liquid G’, ‘Liquid E’ or ‘Liquid Ecstasy’)

Ketamine (also called ‘K’, ‘Special K’ or ‘Vitamin K’)

Poppers (also called ‘Snappers’ or ‘Liquid gold’)

Inhalants (such as glue, nail polish remover, gasoline, solvents, butane, propellants - whippets)

Hallucinogens or psychedelics (also called ‘PCP’, ‘LSD’, ‘Acid’ or ‘Mushrooms’)

Prescription stimulants (also called uppers, ups, pep pills, and include drugs like Adderall and Ritalin)

Prescription painkillers (such as Vicodin, Codeine, Percocet, Hydrocodone, Morphine or OxyContin)

Prescription depressants (such as Ativan, Klonopin, Librium, Valium or Xanax)

Erectile medications (Viagra, Cialis, or Levitra)

Other drug(s): _____________

**Partner alcohol and drug use during sex - past 6 months**

1. **Generally, in the last 6 months, did your partner(s) binge drink alcohol (5 or more alcoholic drinks within 2 hours) two hours before or during sex?**

None of the time

Some of the time

Most of the time

All the time

I don’t know

1. **Generally, in the last 6 months, did your partner(s) take any drugs or get high two hours before or during sex?**

None of your partners

Most of your partners

Some of your partners

All your partners

I don't know

**SKIP Q.I60, IF ANSWER TO Q.I59 IS “NONE OF YOUR PARTNERS”**

1. **In general, which drugs did your partner(s) use two hours before or during sex in the last 30 days? Choose all that apply.**

Marijuana (also called ‘Weed’ or ‘Pot’)

Synthetic marijuana (also called ‘K2’ or ‘Spice’)

Cocaine or crack (also called ‘Coke’, ‘Snow’, ‘Blow’, ‘Rock’ or ‘Freebase’)

Heroin (also called ‘Smack’, ‘Junk’, ‘Black tar’ or ‘China white’)

Fentanyl (also called ‘Apache’, ‘China Girl’, ‘China White,’ ‘Dance Fever’ or ‘Jackpot’)

Ecstasy (also called ‘Molly,’ ‘E,’ or ‘MDMA’)

Methamphetamines (also called ‘Meth’, ‘Crystal meth’, ‘Crank’ or ‘Speed’)

GHB (also called ‘G’, ‘Liquid G’, ‘Liquid E’ or ‘Liquid Ecstasy’)

Ketamine (also called ‘K’, ‘Special K’ or ‘Vitamin K’)

Poppers (also called ‘Snappers’ or ‘Liquid gold’)

Inhalants (such as glue, nail polish remover, gasoline, solvents, butane, propellants - whippets)

Hallucinogens or psychedelics (also called ‘PCP’, ‘LSD’, ‘Acid’ or ‘Mushrooms’)

Prescription stimulants (also called uppers, ups, pep pills, and include drugs like Adderall and Ritalin)

Prescription painkillers (such as Vicodin, Codeine, Percocet, Hydrocodone, Morphine or OxyContin)

Prescription depressants (such as Ativan, Klonopin, Librium, Valium or Xanax)

Erectile medications (Viagra, Cialis, or Levitra)

Other drug(s): _____________

**Condom use in the last 6 months**

For the following questions, please use the scales below where 0% is “Never used a condom” and 100% is “Used a condom all of the time.”

**FOR Q.I61-I63, PIPE IN RESPONSES TO Q.I2-I3**

**In the last 6 months, how often did you use a condom…**

1. **during FRONTAL sex? (your penis in their vagina)** ____________

This does not apply to me

1. **during ANAL insertive sex? (you were the ‘top’)** ____________

This does not apply to me

1. **during ANAL receptive sex? (you were the ‘bottom’)** ____________

**Partners 5 to 10 years older**

1. **In the past six months, how many of your sexual partners were 5-10 years older than you?** None

Number of partners: _____________________

**Partners more than 10 years older**

1. **In the past six months, how many of your sexual partners were more than 10 years older than you?**

None

Number of partners: _____________________

**Partner Type**

1. **What types of partners have you had in the last 6 months? Choose all that apply.**

Primary partner

Consistent casual partner

Single encounter

None

I don’t know

*A* ***primary partner is*** *someone you consider yourself to be in a relationship with (e.g., boyfriend, girlfriend, life partner, etc.).*

*A* ***consistent casual partner*** *is someone you have sex with on a regular basis but do not consider yourself to be in a relationship with (e.g., fuck buddy, friend with benefits, ex-boyfriend).*

*A* ***single encounter*** *is someone you may have met briefly and had sex with but do not intend to have sex with him or her again (e.g., one-night stand, hookup, etc.).*

**SKIP Q.I66-I73, IF RESPONSE TO Q.I66 IS “CONSISTENT CASUAL PARTNER” OR “SINGLE ENCOUNTER” OR “NONE”**

**Primary/Main partners in last 6 months**

1. **In total, how many of your partners in the last six months were PRIMARY/MAIN**

**partners? By primary partner, I mean someone that you consider yourself to be in a relationship with (e.g., boyfriend, girlfriend, life partner, etc.).** ______________________

**Age of Primary partner**

1. **What is the age of your PRIMARY/MAIN partner? If you are having trouble remembering, please provide your best estimate.**

___________

**Ethnicity of Primary Partner**

1. **What is the ethnicity of your PRIMARY/MAIN partner?**

American Indian / Native American

Asian / Asian American / Pacific Islander

Black / African American

Latino / Hispanic

White / Caucasian

Other

Mixed / More than one of the above

I don't know

Refuse to answer

**HIV Status of Primary partner**

1. **What is the current HIV status of your PRIMARY/MAIN partner?**

Positive

Negative

I don't know

Refuse to answer

**SKIP Q.I71-I73, IF RESPONSE TO Q.I70 IS “I DON’T KNOW” OR “REFUSE”**

**Knowledge of HIV Status of Primary Partner**

1. **How do you know your PRIMARY/MAIN partner’s HIV status?**

Partner told you

Partner showed you his test results

HIV tested together

Assumed his status is negative

Other

I don't know

Refuse to answer

**SKIP Q.I72-I73, IF RESPONSE TO Q.I70 IS “NEGATIVE”**

1. **Is your partner in treatment?** No Yes I don’t know
2. **Is your partner…(check all that apply)**

Taking HIV medication

Using alternative treatment(s)

Undetectable (virally suppressed)

Other, please specify: __________________________

I don’t know

**Presence of Sexual Agreement**

The following question asks about your sexual relationship with **YOUR PRIMARY PARTNER**. We would like to learn whether or not you are sexually exclusive or if you have agreements about being with other people.

1. **Which of the following scenarios best describes the sexual agreement that you and your primary partner have?**

We cannot have any sex with an outside partner

We can have sex with outside partners but with some restrictions

We can have sex with outside partners without any restrictions

We do not have an agreement

**Meeting Sexual or Romantic Partners**

1. **In the last 6 months, how were you most likely to meet a sexual or romantic partner? (Check all that apply.)**

Through a friend

Social media site (Facebook, Instagram, etc.)

Dating app (Grindr, Tinder, Scruff, etc.)

Craigslist

At school or work

At a bar or club

Bath houses

At other public sex /cruising places (e.g., parks, sex clubs)

Other (specify): ________________________

Refuse to answer

**Sex exchange**

Many people have exchanged sexual acts or favors for things like money, drugs, or a place to stay. The

next question asks about any of these types of experiences you may have had. Remember that your

answers are completely confidential.

1. **In the last 6 months, have you received any of the following in exchange for sex? (Check**

**all that apply)**

Money

Drugs

A place to stay

Clothes or jewelry

Travel

Job

Car / ride

Food

Hormones

Silicone

Gender-affirming treatments (i.e., voice training, hair removal, Botox)

None

Don't know

Refuse to answer

**SKIP Q.I76, IF ANSWER TO Q.I75 IS “NONE”**

**Sex exchange - no condom**

1. **In these situations, how often were you asked to bareback or to not use a condom?**

Never

Not asked, but that is something expected of me

Rarely

Sometimes

Often

Almost always

Always

Don't know

Refuse to answer

**Perceived control over partners’ use of condoms**

1. **How much control do you have over whether or not your partner uses condoms? Please use the scale below, where 0% is No Control and 100% is Total Control. You can select any point on the scale.**

*Responses were based on a visual analogue scale ranging from `No control’ (0) to `Total control’ (100). Low perceived control over condom use: 0 to 70; High perceived control over condom use: 71 to 100*

**Condom use self-efficacy**

**How strongly do you agree or disagree with the following statements:**

1. **I feel confident in my ability to put a condom on myself or my partner.**

Strongly Disagree
 Disagree

Undecided

Agree

Strongly Agree

1. **I feel confident I could purchase condoms without feeling embarrassed.**

Strongly Disagree
 Disagree

Undecided

Agree

Strongly Agree

1. **I feel confident I could remember to carry a condom with me should I need one.**

Strongly Disagree
 Disagree

Undecided

Agree

Strongly Agree

1. **I feel confident in my ability to discuss condom usage with any partner I might have.**

Strongly Disagree
 Disagree

Undecided

Agree

Strongly Agree

1. **I feel confident in my ability to suggest using condoms with a new partner.**

Strongly Disagree
 Disagree

Undecided

Agree

Strongly Agree

1. **I feel confident I could suggest using a condom without my partner feeling “diseased”.**

Strongly Disagree
 Disagree

Undecided

Agree

Strongly Agree

1. **I feel confident in my ability to persuade a partner to accept using a condom when we have sex.**

Strongly Disagree
 Disagree

Undecided

Agree

Strongly Agree

1. **I feel confident I could use a condom during intercourse without reducing any sexual sensations.**

Strongly Disagree
 Disagree

Undecided

Agree

Strongly Agree

1. **I feel confident I could gracefully remove and dispose of a condom after sexual intercourse.**

Strongly Disagree
 Disagree

Undecided

Agree

Strongly Agree

1. **I would not feel confident suggesting using condoms with a new partner because I would be afraid he or she would think I've had a past homosexual experience.**

Strongly Disagree
 Disagree

Undecided

Agree

Strongly Agree

1. **I feel confident that I would remember to use a condom even after I have been drinking.**

Strongly Disagree
 Disagree

Undecided

Agree

Strongly Agree

1. **I feel confident that I would remember to use condom even if I were high.**

Strongly Disagree
 Disagree

Undecided

Agree

Strongly Agree

1. **I would not feel confident suggesting using condoms with a new partner because I would be afraid he or she would think I have a sexually transmitted disease.**

Strongly Disagree
 Disagree

Undecided

Agree

Strongly Agree

1. **I would not feel confident suggesting using condoms with a new partner because I would be afraid he or she would think I thought they had a sexually transmitted disease.**

Strongly Disagree
 Disagree

Undecided

Agree

Strongly Agree

1. **If I were to suggest using a condom to a partner, I would feel afraid that he or she would reject me.**

Strongly Disagree
 Disagree

Undecided

Agree

Strongly Agree

1. **If I were unsure of my partner's feelings about using condoms I would not suggest using one.**

Strongly Disagree
 Disagree

Undecided

Agree

Strongly Agree

1. **If my partner and I were to try to use a condom and did not succeed, I would feel embarrassed to try to use one again (e.g., not being able to unroll condom, putting it on backwards or awkwardness).**

Strongly Disagree
 Disagree

Undecided

Agree

Strongly Agree

**Section J. Mental Health**

***This section is INTERVIEWER-ADMINISTERED***

**Mindfulness (CARD 24)**

Below is a collection of statements about your everyday experience. Please indicate how frequently or infrequently you currently have each experience. Please answer according to what *really reflects* your experience rather than what you think your experience should be.

|  | **Almost never** | **Very infrequently** | **Somewhat infrequently** | **Somewhat frequently** | **Very frequently** | **Almost always** |
| --- | --- | --- | --- | --- | --- | --- |
| 1. I could be experiencing some emotion and not be conscious of it until some time later. | 1 | 2 | 3 | 4 | 5 | 6 |
| 1. I break or spill things because of carelessness, not paying attention, or thinking of something else. | 1 | 2 | 3 | 4 | 5 | 6 |
| 1. I need to walk quickly to get where I’m going without paying attention to what I experience along the way. | 1 | 2 | 3 | 4 | 5 | 6 |
| 1. I tend not to notice feelings of physical tension or discomfort until they really grab my attention. | 1 | 2 | 3 | 4 | 5 | 6 |
| 1. I forget a person’s name almost as soon as I’ve been told it for the first time. | 1 | 2 | 3 | 4 | 5 | 6 |
| 1. It seems I am “running on automatic,” without much awareness of what I’m doing. | 1 | 2 | 3 | 4 | 5 | 6 |
| 1. I rush through activities without being really attentive to them. | 1 | 2 | 3 | 4 | 5 | 6 |
| 1. I get so focused on the goal I want to achieve that I lose touch with what I’m doing right now to get there. | 1 | 2 | 3 | 4 | 5 | 6 |
| 1. I do jobs or tasks automatically, without being aware of what I’m doing. | 1 | 2 | 3 | 4 | 5 | 6 |
| 1. I find myself listening to someone with one ear, doing something else at the same time. | 1 | 2 | 3 | 4 | 5 | 6 |
| 1. I get to places on ‘automatic pilot’ and then wonder why I went there. | 1 | 2 | 3 | 4 | 5 | 6 |
| 1. I find myself preoccupied with the future or the past. | 1 | 2 | 3 | 4 | 5 | 6 |
| 1. I find myself doing things without paying attention. | 1 | 2 | 3 | 4 | 5 | 6 |
| 1. I snack without being aware that I’m eating. | 1 | 2 | 3 | 4 | 5 | 6 |

**Brief Symptom Inventory (CARD 26)**

Below is a list of problems people sometimes have. Read each one carefully and circle the number of the response that best describes **HOW MUCH THAT PROBLEM HAS**

**DISTRESSED OR BOTHERED YOU DURING THE PAST 7 DAYS INCLUDING TODAY.**

Circle only one number for each problem (0 1 2 3 4).

**0 = Not at all 1 = A little bit 2 = Moderately 3 = Quite a bit 4 = Extremely**

| Faintness or dizziness | 0 | 1 | 2 | 3 | 4 |
| --- | --- | --- | --- | --- | --- |
| 1. Feeling no interest in things | 0 | 1 | 2 | 3 | 4 |
| 1. Nervousness or shakiness inside | 0 | 1 | 2 | 3 | 4 |
| 1. Pains in heart or chest | 0 | 1 | 2 | 3 | 4 |
| 1. Feeling lonely | 0 | 1 | 2 | 3 | 4 |
| 1. Feeling tense or keyed up | 0 | 1 | 2 | 3 | 4 |
| 1. Nausea or upset stomach | 0 | 1 | 2 | 3 | 4 |
| 1. Feeling blue | 0 | 1 | 2 | 3 | 4 |
| 1. Suddenly scared for no reason | 0 | 1 | 2 | 3 | 4 |
| 1. Trouble getting your breath | 0 | 1 | 2 | 3 | 4 |
| 1. Feelings of worthlessness | 0 | 1 | 2 | 3 | 4 |
| 1. Spells of terror or panic | 0 | 1 | 2 | 3 | 4 |
| 1. Numbness or tingling in parts of your body | 0 | 1 | 2 | 3 | 4 |
| 1. Feeling hopeless about the future | 0 | 1 | 2 | 3 | 4 |
| 1. Feeling so restless you couldn’t sit still | 0 | 1 | 2 | 3 | 4 |
| 1. Feeling weak in parts of your body | 0 | 1 | 2 | 3 | 4 |
| 1. Thoughts of ending your life | 0 | 1 | 2 | 3 | 4 |
| 1. Feeling fearful | 0 | 1 | 2 | 3 | 4 |

***THESE MEASURES ARE SELF-ADMINISTERED**

**Self-Injury**

1. **Have you ever purposefully made a cut, burn, or some injury to your body?**

No

Yes

Don’t Know

Refuse to Answer

**SKIP Q.J33, IF RESPONSE TO Q.J32 IS “NO”**

1. **In the past 3 months, how frequently have you made a cut, burn, or some other injury to your body?**

Never

Rarely

Sometimes

Often

Very Often

Don’t Know

Refuse to Answer

**Suicide**

1. **During the past 12 months, did you ever feel so sad or hopeless almost every day for two weeks or more in a row that you stopped doing some usual activities?**

No

Yes

I DON’T KNOW

RF

1. **In the last 12 months, have you seriously considered attempting suicide?**

No

Yes

I DON’T KNOW

RF

1. **In the last 12 months, did you make a plan about how you would attempt suicide?**

No

Yes

I DON’T KNOW
RF

1. **In the last 12 months, how many times have you actually attempted suicide?**

0 times

1 time

2 or 3 times

4 or 5 times

6 or more times

I DON’T KNOW

RF

**SKIP Q.J38, IF ANSWER TO Q.J37 IS “0 TIMES”**

1. **Did any attempt result in an injury, poisoning, or overdose that had to be treated by a doctor or other medical professional?**

No

Yes

I DON’T KNOW

RF

**Stressful events**

**Have you experienced any of the following life events in the LAST 6 MONTHS? If so, please select the number in the scale from 1 to 10 that best describes how 'stressed' you have felt overall.**

1 2 3 4 5 6 7 8 9 10

Not at all Moderately Unbearably

Stressful Stressful Stressful

| **Life Event** | **Experienced in Last 6 Months?** | **Stress Score** |
| --- | --- | --- |
| Family Arguments | Yes No  1 2 | 1 2 3 4 5 6 7 8 9 10 |
| 1. **Arguments with a partner** | Yes No  1 2 | 1 2 3 4 5 6 7 8 9 10 |
| 1. **A minor illness/surgery** | Yes No  1 2 | 1 2 3 4 5 6 7 8 9 10 |
| 1. **A close friend/relative had a serious illness** | Yes No  1 2 | 1 2 3 4 5 6 7 8 9 10 |
| 1. **Your partner died** | Yes No  1 2 | 1 2 3 4 5 6 7 8 9 10 |
| 1. **A close friend/relative died** | Yes No  1 2 | 1 2 3 4 5 6 7 8 9 10 |
| 1. **Your relationship with your partner ended** | Yes No  1 2 | 1 2 3 4 5 6 7 8 9 10 |
| 1. **You were separated from your partner because of relationship difficulties** | Yes No  1 2 | 1 2 3 4 5 6 7 8 9 10 |
| 1. **You were separated from your partner because of reasons other than relationship difficulties** | Yes No  1 2 | 1 2 3 4 5 6 7 8 9 10 |
| 1. **You have had problems/difficulties with a close friend** | Yes No  1 2 | 1 2 3 4 5 6 7 8 9 10 |
| 1. **You failed an exam/test** | Yes No  1 2 | 1 2 3 4 5 6 7 8 9 10 |
| 1. **You dropped out of a training program** | Yes No  1 2 | 1 2 3 4 5 6 7 8 9 10 |
| 1. **You had difficulties with people at work** | Yes No  1 2 | 1 2 3 4 5 6 7 8 9 10 |
| 1. **You experienced difficulties with your employer** | Yes No  1 2 | 1 2 3 4 5 6 7 8 9 10 |
| 1. **You were demoted** | Yes No  1 2 | 1 2 3 4 5 6 7 8 9 10 |
| 1. **You were fired/laid off** | Yes No  1 2 | 1 2 3 4 5 6 7 8 9 10 |
| 1. **You were unemployed, seeking work but unable to find it** | Yes No  1 2 | 1 2 3 4 5 6 7 8 9 10 |
| 1. **There were changes at work that were difficult to handle** | Yes No  1 2 | 1 2 3 4 5 6 7 8 9 10 |
| 1. **You were forced to move out of your house** | Yes No  1 2 | 1 2 3 4 5 6 7 8 9 10 |
| 1. **Something you valued was lost/stolen** | Yes No  1 2 | 1 2 3 4 5 6 7 8 9 10 |
| 1. **You have had financial problems** | Yes No  1 2 | 1 2 3 4 5 6 7 8 9 10 |
| 1. **You have had problems with the police** | Yes No  1 2 | 1 2 3 4 5 6 7 8 9 10 |
| 1. **You were involved in a serious accident** | Yes No  1 2 | 1 2 3 4 5 6 7 8 9 10 |
| 1. **You have had problems associated with your sexuality** | Yes No  1 2 | 1 2 3 4 5 6 7 8 9 10 |
| 1. **You have been hassled or verbally threatened** | Yes No  1 2 | 1 2 3 4 5 6 7 8 9 10 |
| 1. **You have been beaten up or verbally threatened** | Yes No  1 2 | 1 2 3 4 5 6 7 8 9 10 |
| 1. **You found out that someone you know is HIV positive** | Yes No  1 2 | 1 2 3 4 5 6 7 8 9 10 |
| 1. **You found out that someone you know died of AIDS** | Yes No  1 2 | 1 2 3 4 5 6 7 8 9 10 |
| 1. **The health of someone you know who is HIV positive is deteriorating** | Yes No  1 2 | 1 2 3 4 5 6 7 8 9 10 |
| 1. **You have felt that your own health may be deteriorating** | Yes No  1 2 | 1 2 3 4 5 6 7 8 9 10 |
| 1. **Your doctor has told you that your health is deteriorating** | Yes No  1 2 | 1 2 3 4 5 6 7 8 9 10 |
| 1. **You thought you were HIV positive  (HIV- only)** | Yes No  1 2 | 1 2 3 4 5 6 7 8 9 10 |
| 1. **Family member had trouble with the police** | Yes No  1 2 | 1 2 3 4 5 6 7 8 9 10 |
| 1. **Violence in the family** | Yes No  1 2 | 1 2 3 4 5 6 7 8 9 10 |
| 1. **Credit card debt** | Yes No  1 2 | 1 2 3 4 5 6 7 8 9 10 |
| 1. **Family members works long hours** | Yes No  1 2 | 1 2 3 4 5 6 7 8 9 10 |
| 1. **Parents divorced** | Yes No  1 2 | 1 2 3 4 5 6 7 8 9 10 |
| 1. **You came out to your family** | Yes No  1 2 | 1 2 3 4 5 6 7 8 9 10 |
| 1. **Family had financial problems** | Yes No  1 2 | 1 2 3 4 5 6 7 8 9 10 |
| 1. **Increased number of arguments with family members about your gender identity** | Yes No  1 2 | 1 2 3 4 5 6 7 8 9 10 |
| 1. **Trouble with classmates over your gender identity** | Yes No  1 2 | 1 2 3 4 5 6 7 8 9 10 |
| 1. **Losing a close friend because of your gender identity** | Yes No  1 2 | 1 2 3 4 5 6 7 8 9 10 |
| 1. **Increased number of arguments with a close friend over your gender identity** | Yes No  1 2 | 1 2 3 4 5 6 7 8 9 10 |

**Section K. Personality and Attitudes**

***This section is INTERVIEWER-ADMINISTERED***

**Self-Esteem**

The following questions are about your overall level of self-esteem, or how you feel about yourself and your abilities**.**

1. **On the whole, I am satisfied with myself**
   1. Strongly Disagree
   2. Disagree
   3. Agree
   4. Strongly agree
2. **At times, I think I am no good at all**
   1. Strongly Disagree
   2. Disagree
   3. Agree
   4. Strongly agree
3. **I feel that I have a number of good qualities**
   1. Strongly Disagree
   2. Disagree
   3. Agree
   4. Strongly agree
4. **I am able to do things as well as most other people**
   1. Strongly Disagree
   2. Disagree
   3. Agree
   4. Strongly agree
5. **I feel I do not have much to be proud of**
   1. Strongly Disagree
   2. Disagree
   3. Agree
   4. Strongly agree
6. **I certainly feel useless at time**
   1. Strongly Disagree
   2. Disagree
   3. Agree
   4. Strongly agree
7. **I feel that I'm a person of worth, at least on an equal plane with others**
   1. Strongly Disagree
   2. Disagree
   3. Agree
   4. Strongly agree
8. **I wish I could have more respect for myself**
   1. Strongly Disagree
   2. Disagree
   3. Agree
   4. Strongly agree
9. **All in all, I am inclined to feel that I am a failure**
   1. Strongly Disagree
   2. Disagree
   3. Agree
   4. Strongly agree
10. **I take a positive attitude toward myself**
    1. Strongly Disagree
    2. Disagree
    3. Agree
    4. Strongly agree

**Optimism: The Life Orientation Test (CARD 8)**

For the next set of questions, please be as honest and accurate as you can throughout. Try not to let your response to one statement influence your responses to other statements. There are no "correct" or "incorrect" answers. Answer according to your own feelings, rather than how you think "most people" would answer.

1. **In uncertain times, I usually expect the best.**

I disagree a lot

I disagree a little

I neither agree nor disagree

I agree a little

I agree a lot

1. **It's easy for me to relax.**

I disagree a lot
I disagree a little
I neither agree nor disagree
I agree a little
I agree a lot

1. **If something can go wrong for me, it will.**

I disagree a lot
I disagree a little
I neither agree nor disagree
I agree a little
I agree a lot

1. **I'm always optimistic about my future.**

I disagree a lot
I disagree a little
I neither agree nor disagree
I agree a little
I agree a lot

1. **I enjoy my friends a lot.**

I disagree a lot
I disagree a little
I neither agree nor disagree
I agree a little
I agree a lot

1. **It's important for me to keep busy.**

I disagree a lot
I disagree a little
I neither agree nor disagree
I agree a little
I agree a lot

1. **I hardly ever expect things to go my way.**

I disagree a lot
I disagree a little
I neither agree nor disagree
I agree a little
I agree a lot

1. **I don't get upset too easily.**

I disagree a lot
I disagree a little
I neither agree nor disagree
I agree a little
I agree a lot

1. **I rarely count on good things happening to me.**

I disagree a lot
I disagree a little
I neither agree nor disagree
I agree a little
I agree a lot

1. **Overall, I expect more good things to happen to me than bad.**

I disagree a lot
I disagree a little
I neither agree nor disagree
I agree a little
I agree a lot
